# Mesoporous silica nanoparticle-encapsulated *Bifidobacterium* attenuates brain Aβ burden and improves olfactory dysfunction of APP/PS1 mice by nasal delivery

**DOI:** 10.1186/s12951-022-01642-z

**Published:** 2022-10-07

**Authors:** Ni Liu, Changwen Yang, Xiaohan Liang, Kai Cao, Jun Xie, Qingming Luo, Haiming Luo

**Affiliations:** 1 Britton Chance Center for Biomedical Photonics, Wuhan National Laboratory for Optoelectronics, Huazhong University of Science and Technology, Wuhan, 430074 Hubei China; 2 MoE Key Laboratory for Biomedical Photonics, School of Engineering Sciences, Huazhong University of Science and Technology, Wuhan, China; 3 School of Biomedical Engineering, Hainan University, Haikou, 570228 Hainan China

**Keywords:** Alzheimer’s disease, Nasal delivery, *Bifidobacterium*, β-amyloid, Mesoporous silica nanospheres (MSNs)

## Abstract

**Background:**

Dysbiosis or imbalance of gut microbiota in Alzheimer's disease (AD) affects the production of short-chain fatty acids (SCFAs), whereas exogenous SCFAs supplementation exacerbates brain Aβ burden in APP/PS1 mice. *Bifidobacterium* is the main producer of SCFAs in the gut flora, but oral administration of *Bifidobacterium* is ineffective due to strong acids and bile salts in the gastrointestinal tract. Therefore, regulating the levels of SCFAs in the gut is of great significance for AD treatment.

**Methods:**

We investigated the feasibility of intranasal delivery of MSNs-*Bifidobacterium* (MSNs-Bi) to the gut and their effect on behavior and brain pathology in APP/PS1 mice.

**Results:**

Mesoporous silica nanospheres (MSNs) were efficiently immobilized on the surface of *Bifidobacterium*. After intranasal administration, fluorescence imaging of MSNs-Bi in the abdominal cavity and gastrointestinal tract revealed that intranasally delivered MSNs-Bi could be transported through the brain to the peripheral intestine. Intranasal administration of MSNs-Bi not only inhibited intestinal inflammation and reduced brain Aβ burden but also improved olfactory sensitivity in APP/PS1 mice.

**Conclusions:**

These findings suggested that restoring the balance of the gut microbiome contributes to ameliorating cognitive impairment in AD, and that intranasal administration of MSNs-Bi may be an effective therapeutic strategy for the prevention of AD and intestinal disease.

**Supplementary Information:**

The online version contains supplementary material available at 10.1186/s12951-022-01642-z.

## Introduction

Alzheimer's disease (AD) is a multifactorial and irreversible progressive neurodegenerative disease associated with cognitive impairment and memory loss. The ‘‘pathogen hypothesis’’ in AD suggests that gut microbiota dysbiosis is associated with the development of AD [1–3]. Dysregulation of the gut microbiota may increase the permeability of the gut and the blood–brain barrier (BBB) [4]. Alterations in permeability can promote gut microbiota-derived molecules (e.g. lipopolysaccharides, LPS) and metabolites (e.g. short-chain fatty acids, SCFAs; secondary bile acids, BAs) to influence neuroinflammatory and immune responses, thereby affecting brain function [5]. Among gut microbiota-derived metabolites, SCFAs have beneficial effects on brain function through immune, endocrine, vagal, and other humoral pathways [6, 7]. For example, SCFAs can maintain immune homeostasis by ameliorating intestinal barrier dysfunction [5, 8]. SCFA in AD is lower than in normal mice, and exogenous SCFA supplementation in turn exacerbated Aβ plaque deposition and microglial damage in the brains of APP/PS1 transgenic mice [9]. The probiotic *Bifidobacterium* is one of the main producers of SCFAs in the intestinal flora and is involved in regulating the balance of intestinal flora [10]. Therefore, modulating SCFA levels by increasing the abundance of *Bifidobacterium* in the gut microbiota has important implications for the treatment and intervention of AD.

How to effectively deliver *Bifidobacterium* to the gut to increase the abundance of *Bifidobacterium* is a current challenge. Oral *Bifidobacterium* has received great attention. However, oral administration of *Bifidobacterium* is ineffective due to strong acidity and bile salts in the gastrointestinal tract [11, 12]. Bile salts cause leakage of ions and other cellular components, leading to the death of *Bifidobacterium* [13]. Studies have shown that *Bifidobacterium* can only survive for 3 h when the bile salt concentration is maintained at around 0–1.5% [14]. Although encapsulation of probiotics [15] or into tablets and drops can improve the survival rate of probiotics in the gastrointestinal tract [16], their therapeutic effect is still inevitably limited by impaired probiotic activity. In addition, the stress tolerance of *Bifidobacterium* strains has been improved by genetic modification, but this method is difficult for clinical translation due to the introduction of pathogenic genes [17]. Therefore, there is an urgent need to develop novel *Bifidobacterium* delivery technologies for the intervention and treatment of AD.

Nasal administration is an alternative, non-invasive, and painless technique that has been successfully used in AD treatment [18–21]. However, current nasal administration is limited to the use of various nanocarriers [22, 23], such as liposomes [24], antibodies [25], polymeric nanoparticles [26], and inorganic nanoparticles [27], for drug delivery to the brain [28]. Given the bidirectional communication between the brain and gut microbiota, is it possible to deliver *Bifidobacterium* to the gastrointestinal tract via nasal administration? Mesoporous silica nanoparticles (MSNs) are relatively safe porous materials with good biocompatibility and large pore volume, so they are widely used as drug carriers [29]. Recent studies have shown that MSNs have a remarkable loading capacity for bacteria, and bacteria as nanoparticle carriers can enhance tissue penetration [30, 31]. Therefore, we proposed the hypothesis that intranasal delivery of MSN-encapsulated *Bifidobacterium* to the mouse gut could modulate the levels of intestinal SCFAs, thereby slowing AD pathology. Based on this hypothesis, we characterized synthetic MSNs-*Bifidobacterium* (MSNs-Bi) using Cy3-MSNs and FITC-labeled *Bifidobacterium*, investigated their feasibility for long-distance transport from the nasal cavity to the gut, and evaluated their effects on improving AD pathology. This strategy of balancing gut microbiota by nasal delivery of *Bifidobacteria* into the gut reduces brain Aβ burden and improves cognitive performance in APP/PS1 mice, opening a new avenue for AD therapy.

## Materials and methods

### Antibodies and reagents

An anti-Aβ (6E10) antibody was purchased from Invitrogen (Carlsbad, CA, USA). Anti-Iba1 (019-19,741) was acquired from Wako laboratory chemicals (Osaka, Japan). Pierce streptavidin-coupled Poly-Horseradish Peroxidase (HRP) and protein markers were ordered from Thermo Fisher Scientific (Ann Arbor, MI, US). Thioflavin S (ThioS), dimethyl sulfoxide (DMSO), and bovine serum albumin (BSA) were obtained from Sigma-Aldrich (Shanghai, China). Casein tryptone, yeast extract, and agar were ordered from Sinopharm (Shanghai, China). *Bifidobacterium* selective (BS) culture medium and trypticase-phytone-yeast (TPY) Broth were ordered from Hopebio (Qingdao, China). Cy3-N-hydroxysuccinimide (NHS) ester was purchased from Lumiprobe (Hannover, Germany). The 4′,6-diamidino-2-phenylindole (DAPI) solution was ordered from Beyotime Biotechnology (Shanghai, China), tetrahydrofuran (THF), N-cetyltrimethylammonium bromide (CTAB, 99%) and methanol were purchased from Aladdin (Shanghai, China). Tetraethyl orthosilicate (TEOS, 98%) and paraformaldehyde (PFA) were obtained from Macklin (Shanghai, China). FITC-d-Lys was obtained from Shengguang Biotechnology Co., Ltd. (Xiamen, China). All additional chemicals were purchased from commercial suppliers and used as received.

### Animals

4 month-old transgenic APPSwe/PS1dE9 (APP/PS1) mice were purchased from the Beijing Huafukang Biotechnology Co., Ltd (Beijing, China). Age-matched C57BL/6J mice were purchased from Shulaibao Biotech Co. (Wuhan, China). This study used APP/PS1 and wild-type C57BL/6J mice aged approximately 4–8 months. All mice were maintained under standard specific pathogen-free (SPF) conditions with an ambient temperature of 23 ± 2 °C, air humidity of 40–70%, and an artificial 12 h light/dark cycle. All experiments were approved by the Institutional Animal Care and Use Committee of Huazhong University of Science and Technology.

### Synthesis of MSNs

MSNs were prepared using the coprecipitation method [32]. Briefly, 40 mL of methanol was mixed with 110 mL of ultrapure water, and the pH was adjusted to 11 with ammonia. CTAB was added and heated. When the temperature reached 80 °C, a certain amount of TEOS was added dropwise and stirred vigorously for 3 h. After cooling to room temperature, the solution was centrifuged at 9500 rpm for 10 min, washed 3 times with deionized water, and then vacuum dried overnight to obtain a white solid powder. Finally, the dry white powder was dissolved in a mixed solution of 100 mL of absolute ethanol and 10 mL of concentrated hydrochloric acid. The solution was refluxed for 24 h to remove CTAB. The mass ratio of TEOS/CTAB was controlled at 2:1 to synthesize MSNs. Next, MSNs were mixed with Poly-L-Lysine in a shaker (55 rpm) at 37 °C for 30 min and then vacuum dried to obtain a discolored powder.

### MSNs characterization

Here, 20 μL of 1 mg/mL MSNs was dropped on a copper grid, and excess liquid was removed using filter papers. After drying at room temperature, the morphology of the nanomaterials was observed by transmission electron microscopy (TEM) on a HITACHI HT7700 transmission electron microscope (TEM; Hitachi High-Tech, Japan) at a voltage of 120 kV. Next, dynamic light scattering (DLS; photon correlation spectroscopy) and the Zetasizer Nano-ZS90 system (Malvern Instruments, Worcestershire, UK) were used to measure the size distribution and surface potential of MSNs.

### Fluorescence modification of MSNs

MSNs (10 mg) were dissolved in 1 mL of PBS solution. 5 μL of Cy3-NHS (100 μg) in DMSO solution was slowly added to the MSN suspension, stirred overnight in the dark, followed by centrifugation (5000 rpm, 10 min) to remove unloaded Cy3 from the outer surface of the MSNs.

### FITC-d-Lys staining of *Bifidobacterium *in vitro

*Bifidobacterium* was purchased from the Culture Collection Center of Yunnan Institute of Microbiology. *Bifidobacterium* was grown in the BS culture medium at 37 °C until OD600 reached 0.6. The medium was diluted to an OD600 of 0.3 with fresh medium containing FITC-d-Lys (0.1 mM). The diluted bacteria were further incubated at 37 °C until the OD600 was 1.0–1.5. The bacteria were centrifuged and washed 3 times with the BS culture medium.

### Preparation and characterization of MSNs-Bi

The cultured FITC-labeled *Bifidobacterium* was centrifuged at 8000 ×g for 10 min at 4 °C, then the pellet was washed and resuspended in 5 mL of physiological saline. Then, 1 mL of 40 µg/mL Cy3-MSNs was mixed with 1 mL of FITC labeled-*Bifidobacterium* culture (2.31 ×10^11^ CFU/mL) for 30 min incubation at 37 °C in a shaker (255 rpm). After washing 3 times with PBS, FITC labeled-*Bifidobacterium* and MSNs-Bi were suspended with 2.5% glutaraldehyde solution at 4 °C for 10 h, and then dehydrated in increasing series of water–ethanol solutions (35%, 50%, 70%, 85%, 95%, and 100%), and finally vacuum dried. The morphology of MSNs-Bi and *Bifidobacteri*um was examined by scanning electron microscope (SEM; Sirion 200, the Netherlands).

### In vitro stability of MSNs-Bi

To evaluate the stability of MSNs-Bi, on the one hand, the change of fluorescence intensity of Cy3-MSNs-Bi with time was detected. Briefly, 20 μg Cy3-MSNs-Bi were incubated in 200 μL Krebs–Henseleit solution (D-glucose 2.0 g/L, magnesium sulfate 0.141 g/L, potassium phosphate monobasic 0.16 g/L, potassium chloride 0.35 g/L, sodium chloride 6.9 g/L, calcium chloride dihydrate 0.373 g/L, and sodium bicarbonate 2.1 g/L) at 37 °C for 0, 0.5, 1, 2, 3, 4, 6, 9, and 12 h. The Cy3 fluorescence signals of samples were detected using a multiscan FC microplate photometer (Thermos, USA) at an emission wavelength of 490 nm (excitation at 440 nm). The release curve of Cy3 in MSNs is shown in Additional file 1: Fig. S10, and the Cy3 fluorescence intensity of MSNs-Bi will superimpose the free release amount of Cy3 from MSNs.

On the other hand, the activity of *Bifidobacterium* at different time points was detected. 1 μL of MSNs-Bi at different time points was mixed with 100 μL of culture medium, and their activity was evaluated by counting the number of colonies formed by *Bifidobacterium* on the culture medium.

### Effects of MSNs on bacterial proliferation

Antimicrobial kinetics were evaluated using a co-culture assay [15]. Briefly different concentrations (5, 10, 40, 60, 80, 100, and 160 μg/mL) of MSNs were added to 10 mL of *Escherichia coli* (*E. coli*) and *Bifidobacterium* cultures (1 ×10^5^ CFU/mL). Subsequently, the *E. coli* and *Bifidobacterium* cultures with MSNs were shaken at 37 °C for 12 h. At each time interval, 100 μL of the medium was transferred to a 96-well plate, and the optical density at 600 nm was measured with a NanoDrop 2000 (Thermo Scientific, USA) and the minimal inhibitory concentration was calculated.

To determine the minimum bactericidal concentration (MBC) of MSNs, 100 μL of *E. coli* and *Bifidobacterium* cultures (1 ×10^5^ CFU/mL) were inoculated on Luria Broth (LB) agar or BS culture medium pretreated with different concentrations of MSNs (5, 10, 40, 60, 80, 100, and 160 μg/mL) overnight at 37 °C. After 24 h, colonies were observed and digital images of each plate were captured to evaluate the effect of MSNs.

### Bacterial activity test of MSNs-*E. coli* and MSN-Bi in gastrointestinal stimulated fluid

To determine the release characteristics of *E. coli* and *Bifidobacterium* loaded with MSNs under physiological conditions of the gastrointestinal tract, simulated gastric fluid (SGF) and simulated intestinal fluid (SIF) were prepared according to the method specified in United States Pharmacopoeia. Briefly, SGF was prepared by dissolving 2 g/L NaCl and 3.2 g pepsin (350 activity units per mg) to make a final volume of 1 L ddH_2_O with a pH of 2.0. The SIF at pH 7.2 was prepared using 0.05 mol/L KH_2_PO4 and 1.2% (w/v) bile salts. Then, 100 μL of MSN, MSNs*-E. coli*, and MSNs-Bi (1 × 10^5^ CFU/mL) were cultured in 1 mL of SGF and SIF at 37 °C for 5, 30, 60, and 120 min. The number of colonies formed by *E. coli* and *Bifidobacterium* on LB agar and BS culture medium was used to evaluate the effect of MSNs on *Bifidobacterium* viability.

### Fluorescence imaging and biodistribution studies of MSNs-Bi

To evaluate the biodistribution of MSNs-Bi, 4 month-old male C57BL/6J mice (n = 3) were intranasally injected with MSNs-Bi (1 ×10^9^ CFU/mL). An equal volume of PBS was used as a control. Major organs (e.g. abdomen, brain, lung, spinal cord, intestine, and intestinal contents) were collected, washed with PBS, and imaged using a homemade whole-body imaging system at 5 h post-injection. In addition, tissues of the brain, abdomen, spine, and intestine were fixed in 4% (w/v) PFA overnight and sliced in 50 μm sections using a CM1950 Leica cryostat (Leica Biosystems, Germany). Fluorescence signals of Cy3-MSNs and FITC-labeled *Bifidobacterium* in tissues were observed by confocal imaging.

### In vivo tracking of *Bifidobacterium*

To evaluate the superiority of nasally administered MSNs-Bi over conventional oral administration of *Bifidobacterium*, 4 month-old male C57BL/6J mice (n = 3) were intranasally injected with Cy3-MSNs-Bi (1 ×10^9^ CFU/mL; *Bifidobacteria* were labeled with FITC). An equal amount of FITC-labeled *Bifidobacterium* was administered orally as a control. Then, fresh intestinal contents (stomach, small intestine, and large intestine) were collected and imaged using a homemade whole-body imaging system at 0, 0.25, 0.5, 3, 6, and 9 h post-injection [33, 34].

### Assessing the effect of MSNs-Bi on intestinal inflammation

To evaluate whether intranasal instillation of MSNs-Bi was more effective in alleviating intestinal inflammation in APP/PS1 mice than gavage with *Bifidobacterium*, 4 month-old APP/PS1 mice were treated with PBS, MSNs, MSNs-Bi, and *Bifidobacterium*, respectively. The *Bifidobacterium* were dissolved in PBS before treatment and administered at a dose of 1 ×10^9^ CFU/kg every 4 days for a total of 7 times. After treatment, mice were allowed to rest for 2 months. Then, the mice were sacrificed. Treatment effects were evaluated according to neuroinflammation, brain and intestinal Aβ plaques, intestinal flora, and behavioral status.

### Histopathological analysis

After treatment with PBS, MSNs, *Bifidobacterium*, and MSNs-Bi, colons were collected from mice and then fixed in 4% (w/v) PFA solution overnight. Colon tissues were embedded in paraffin sections and stained with hematoxylin and eosin (H&E). H&E stained sections were imaged on a Nikon Ni-E microscope (Nikon, Minato, Tokyo, Japan). Colonic crypt length in mice treated with PBS, MSNs, *Bifidobacterium*, and MSNs-Bi was evaluated using ImageJ software.

### 16 s DNA library preparation and metagenomic analysis

After collecting feces from APP/PS1 and C57BL/6 mice treated with MSNs, MSNs-Bi, and *Bifidobacterium*, the total DNA of fecal samples was extracted using a high-throughput DNA isolation kit (Boao Classic, Beijing). To prepare the 16 s rDNA library, primers (forward: CCTAYGGGRBGCASCAG; reverse: GGACTACNNGGGTATCTAAT) were utilized to amplify the V3–V4 region of the 16 s rRNA. According to the manufacturer’s protocol, sequencing was performed on the Illumina HiSeq 2500 platform. Furthermore, quality control was carried out using QIIME pipelines. Raw reads were filtered and trimmed using default settings. Subsequently, the pair-end reads were combined and the primers were removed. Next, the taxonomy was assigned by search with a similarity threshold of 97%. Downstream analysis was performed using the R packages ‘MicrobiotaProcess’ and ‘ggplot2’. Alpha diversity is measured based on four different metrics, including Chao1, Abundance-based Coverage Estimator (ACE), Shannon, and Simpson. The differential abundance of the microbiome was calculated using LEfSe of the R package ‘lefser’.

### Immunoprecipitation and western blotting

After treatment with PBS, MSNs, *Bifidobacterium*, and MSNs-Bi, APP/PS1 mice were anesthetized with 0.4 mL Avertin (25 mg/mL) and perfused transcardially with 1 M PBS for 30 min; the tissues (brain, stomach, duodenum, jejunum, ileum, caecum, and colon) were stored at − 80 °C before analysis. Frozen tissues were homogenized in liquid nitrogen and dissolved in tris-buffered saline solution (TBS, 20 mmol/L Tris and 137 mmol/L NaCl, pH 7.6) as previously described [1, 35]. Briefly, the homogenized supernatant was centrifuged (10,000 g, 30 min) at 4 °C to obtain TBS-soluble proteins. The supernatant was aliquoted and stored at –80 °C before analysis. Additionally, 100 μLof brain homogenate supernatant or blood was incubated with 40 μg/mL of 6E10-conjugated protein A/G magnetic beads for 30 min at room temperature. Magnetic beads were washed 5 times with PBS on a magnetic stand. Then, 20 μL of PBS and 4 μL of loading buffer (Boster Biotech, USA) were added and the mixture was boiled at 95 °C for 10 min. Finally, beads were loaded and subjected to 12% tris-tricine sodium dodecyl sulfate–polyacrylamide gel electrophoresis (SDS-PAGE). Proteins separated by SDS-PAGE were transferred onto polyvinylidene fluoride (PVDF) membranes, which were blocked with PBS-T containing 5% skimmed milk for 30 min. Subsequently, membranes were incubated with primary antibody 6E10 (1:1000, 5% skimmed milk) overnight at 4 °C and washed with PBS-T. Next, the membranes were incubated with a second antibody HRP-conjugated goat anti-mouse IgG (H +L) (1:4000, PBS-T) for 1 h. Finally, immunoreactive proteins were identified using ECL substrates (Vazyme, China) on a Tanon 5200 Multi (Shanghai, China).

### Immunofluorescence analysis of tissue sections

Brain, stomach, duodenum, jejunum, ileum, caecum, and colon tissues were collected from APP/PS1 mice treated with intranasal instillation of PBS, MSNs, MSNs-Bi, and *Bifidobacterium*. Next, the tissues were pipetted into 4% (w/v) PFA overnight and then dehydrated in 30% (w/v) sucrose solution. Tissues were then cut into 25 μm sections using a cryo-Leica CM3050S microtome and mounted on adhesion microscope slides (Jiangsu, China). Sections were blocked with 3% BSA for 2 h, treated with 0.2% Triton X-100 (Macklin, China) for 30 min at room temperature, and incubated overnight at 4 °C with the anti-Aβ primary antibody 6E10 (1:1000). Subsequently, sections were incubated with Cy3-conjugated goat anti-mouse IgG (H +L). Finally, all tissue sections were counterstained with 0.002% ThioS solution for 30 min and washed 3 times for 5 min each in 50% ethanol (v/v). Confocal images were acquired on a Zeiss LSM710 at 10 ×magnification and analyzed by Image J software.

### Quantitative analysis of Aβ and inflammatory factors

For quantitative analysis of Aβ and inflammatory factors, frozen tissues of APP/PS1 mice treated with PBS, MSNs, MSNs-Bi, and *Bifidobacterium* were homogenized in liquid nitrogen and extracted using TBS solution. The homogenized supernatant was then centrifuged at 10,000 g for 30 min at 4 °C to obtain TBS-soluble proteins. Following this, Aβ levels in the mouse brain and intestine were quantified using the Aβ_42_ human ELISA kit (Catalog #KHB3441, Invitrogen). The BD Cytometric Bead Array (CBA) Mouse Th1/Th2/Th17 Cytokine kit (Catalog #560485, BD Biosciences) was used to quantify the levels of inflammatory factors in the blood.

### Determination of SCFAs

Feces from different groups of APP/PS1 mice treated with PBS, MSNs, MSNs-Bi, and *Bifidobacterium* were collected and stored at − 80 °C before analysis. Total SCFAs were extracted from 50 mg stool samples using the Mouse Short-Chain Fatty Acid ELISA Kit (Catalog #RJ17989, Renjie Biosciences). Data are expressed as mean content (pg/g) ± SD.

### Behavioral analysis

#### Odor cross-habituation test

To assess the olfactory function of mice after different treatments, an odor cross-habituation test was performed using a previously described method to detect olfactory defects in mice [36]. Monomolecular odorants including heptanone, isoamyl acetate, ( +) enantiomer of limonene, and ethyl valerate (Sigma Aldrich, China) were diluted to 1 ×10^−3^ mol/L in mineral oil and coated on a cotton ball. The cotton ball was then wrapped in an odorless plastic bag with small holes and placed in the bottle cap to prevent the odor liquid from reaching the cage or animals, but allow the volatile odor fumes to diffuse. Odor cross-habituation was tested in four 20 s trials by placing a cotton ball on the side of the animal's cage with a 30 s interval between them. During sniffing, the time recording was terminated when the mouse's nose was within 1 cm of the odor of the cotton ball.

#### Nest construction assay

Nesting behavior is a simple and versatile behavior test that has been widely used to assess motor deficits. Consistently sized paper towels (5 cm ×5 cm) were placed in the same position in the mouse cages in the PBS, MSNs, *Bifidobacterium*, and MSNs*-*Bi treatment groups. After 24 h, the position of the paper towels within the nest was observed.

#### Step down test

During the training phase, mice were initially placed individually in a shock chamber to freely examine the environment for 3–5 min. Then, mice received unconditioned stimulation (0.4 mA). The time the mice jumped to the safety station when it first received an electric shock was considered to be its perception ability. Next, mice were placed in a shock chamber for 30 s to measure immediate response, and the process was repeated 4 times. After 24 h, each mouse was returned to the same safety station in the shock chamber, and the total time and number of shocks within 5 min were recorded.

### Statistical analysis

Statistical analyses were performed using GraphPad Prism version 8.0 for Windows. Data are presented as mean ± standard error of the mean (SEM). One-way or two-way analysis of variance (ANOVA) was used for multiple group comparisons. Statistical significance is indicated in the graph with *p < 0.05, **p < 0.01, ***p < 0.001, ****p < 0.0001, and n.s. (means no significance).

## Results

### Synthesis and characterization of MSNs-Bi

**Fig.1:**
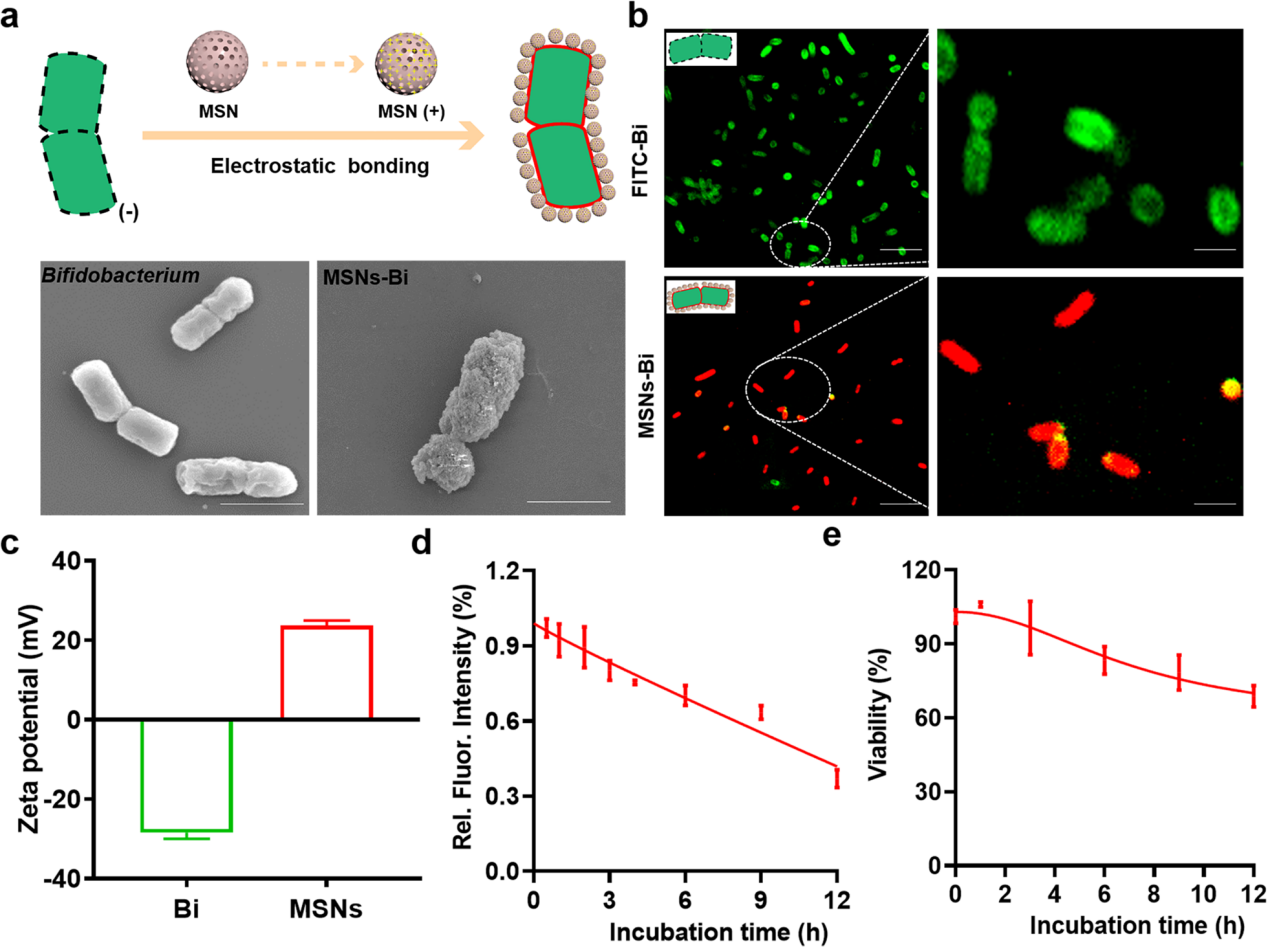
Characterization of MSNs-Bi. **a** The upper panel shows the schematic diagram of MSNs-Bi; the lower panel shows the SEM micrographs of naked *Bifidobacterium* (left) and *Bifidobacterium* with MSNs attached on their surface (right), scale bars = 500 nm. **b** Confocal images of MSNs-Bi synthesized by FITC-labeled *Bifidobacterium* and Cy3-labeled MSNs; FITC (green), Cy3 (Red), scale bars = 5 µm. **c** Zeta potential of *Bifidobacterium* and MSNs. **d** Relative fluorescence intensity of Cy3-MSNs-Bi for monitoring the changes of MSNs on the surface of *Bifidobacterium* in Krebs–Henseleit solution. **e** Viability of *Bifidobacterium* in MSNs-Bi during exposure to Krebs–Henseleit solution

An MSNs-based bacterial brain–gut delivery system was constructed to modulate the levels of intestinal SCFAs by targeted delivery of *Bifidobacterium*, thereby improving intestinal inflammation. TEM and DLS results showed that the synthesized MSNs had an average diameter of 98 ± 10 nm (Additional file 1: Fig. S1). Furthermore, DLS results showed that the zeta potential of *Bifidobacterium* and poly-L-lysine surface-modified MSNs were − 28.48 ± 1.56 and + 23.71 ± 1.24 mV, respectively, which enabled them to form MSNs-Bi through electrostatic bonding (Fig. 1c). In addition, SEM showed uniform distribution of MSNs on the surface of *Bifidobacterium* (Fig. 1a). Confocal imaging and spectrum measurements of MSNs-Bi prepared from Cy3-MSN with FITC-labeled *Bifidobacterium* showed strong Cy3 signals and weak FITC signals (Fig. 1b; Additional file 1: Fig. S2), demonstrating that MSNs were successfully immobilized on the surface of *Bifidobacterium*. Furthermore, to study the stability of MSNs-Bi, MSNs-Bi was incubated with simulated body fluids (Krebs − Henseleit solution) at 37 °C for 12 h, and the stability of MSNs was evaluated by the change of Cy3 fluorescence intensity at different time points. The results showed that the Cy3 fluorescence intensity in MSNs-Bi decayed slowly (Fig. 1d). Moreover, the survival rate of MSN-loaded *Bifidobacterium* did not decrease significantly, and the survival rate was about 70% after 12 h incubation (Fig. 1e; Additional file 1: Fig. S3), further confirming the good stability of MSNs-Bi.

### Evaluation of the activity of *Bifidobacterium* encapsulated in MSNs

**Fig. 2:**
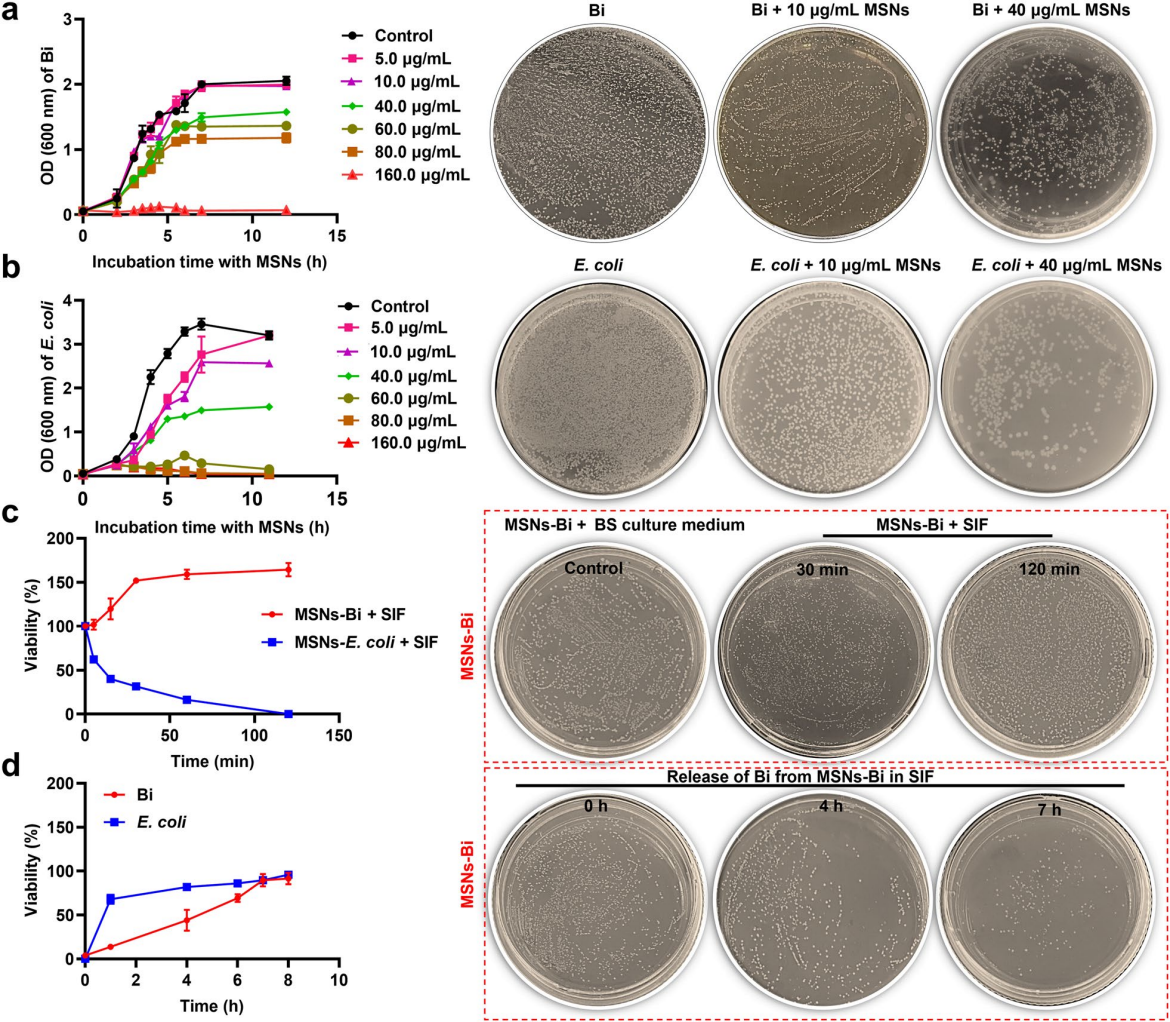
Effects of MSNs on the growth of *Bifidobacterium*. Changes in bacterial growth of (**a)** *Bifidobacterium* and (**b)** *Escherichia coli* (*E. coli*) in the presence of different MSN concentrations. **c** Viability of encapsulated *Bifidobacterium* and *E. coli* during exposure to simulated intestinal fluid (SIF) with bile salts. **d** Release of *Bifidobacterium* and *E. coli* from MSNs encapsulation in SIF at 37 °C. Viability represents the percentage of bacteria surviving relative to the initial population. Data are presented as mean ± SD, n = 3

To evaluate the effect of MSNs on *Bifidobacterium* growth, *Bifidobacterium* was cultured with different concentrations of MSNs in a TPY medium, and then the growth curves of *Bifidobacterium* were then measured. *Bifidobacterium* is a Gram-positive bacteria, thus the Gram-negative bacteria *E. coli* was selected as a control. The results showed that when *Bifidobacterium* was co-cultured with MSN at a concentration lower than 10 µg/mL for 12 h, the growth rate of *Bifidobacterium* was not significantly (*P* = 0.9999) different from that in the untreated group (Fig. 2a). However, the growth rate of *E. coli* was significantly different (*P* < 0.0001) from that in the untreated group when *E. coli* was co-cultured with MSNs at a concentration of 5 µg/mL for 5–8 h (Fig. 2b). With the increase of MSN concentration, MSNs showed a certain inhibitory effect on the growth of *Bifidobacterium* and *E. coli*, but at the same concentration of MSNs, *E. coli* was more sensitive to MSNs than *Bifidobacterium*, which is due to the thicker peptidoglycan layer of *Bifidobacterium*. In addition, bacterial kinetic curves showed a dose- and time-dependent effect of MSNs on the growth of loaded *Bifidobacterium* and *E. coli*, consistent with the colony formation inhibition observed on LB/BS-agar plates (Fig. 2a–b). The minimum bactericidal concentration of *Bifidobacterium* and *E. coli* loaded in MSNs was determined to be 40 µg/mL. Furthermore, the MSN loading rates for *Bifidobacterium* and *E. coli* were 27.13 ± 4.95% and 28.18 ± 2.08%, respectively (Additional file 1: Fig. S4a–b). These results indicated that 40 µg/mL of *Bifidobacterium* was the optimal loading concentration for MSNs.

Studies have shown that encapsulating probiotics in microcapsules can significantly improve their viability in SIF [19]. Figure 2c and Additional file 1: Fig. S4c show the survival rates of *Bifidobacterium* and MSNs-Bi over time. The results showed that the survival rate of *Bifidobacterium* in bile salt SIF at 37 °C decreased sharply and dropped to zero within 10 min. In addition, the survival rate of *E. coli* was similar to that of *Bifidobacterium* under the same conditions (Additional file 1: Fig. S4c–d). In contrast, the survival of *Bifidobacterium* loaded into MSNs increased slowly over time, whereas that of the MSN-*E. coli* group declined slowly and reached zero within 100 min (Fig. 2c). These results indicated that MSNs effectively improved the survival rate of *Bifidobacterium* in SIF. Next, Fig. 2d shows the release of *Bifidobacterium* and *E. coli* from MSNs in bile salt-free SIF over time at 37 °C. The results indicated that in SIF, almost 90% of *Bifidobacterium* and *E. coli* loaded into MSNs were released within 5–7 h, indicating that MSNs indeed delay the release of loaded *Bifidobacterium* and *E. coli*.

### Biodistribution and bioactivity evaluation of nasally delivered MSNs-Bi

**Fig.3:**
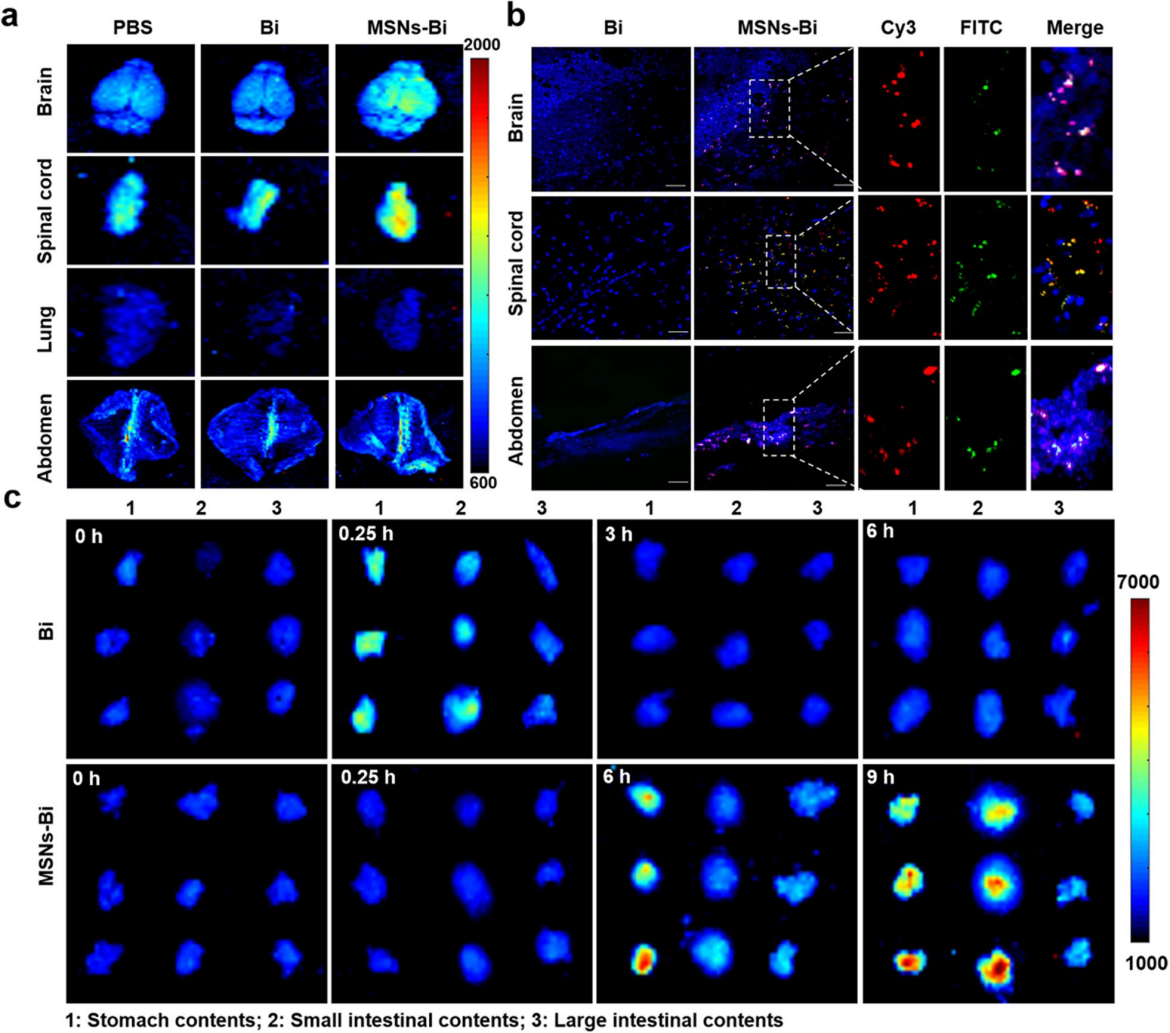
Biodistribution of MSNs-Bi after intranasal instillation. **a** Fluorescence imaging of organs at 5 h after intranasal instillation of MSNs-Bi (tracing FITC-labeled *Bifidobacterium*). **b** Representative confocal images of the brain, spinal cord, and abdomen at 5 h after intranasal instillation of MSNs-Bi; DAPI (blue), FITC (green), and Cy3 (Red); scale bar = 200 μm. **c** Fluorescence imaging of gastrointestinal contents at different time points after oral administration of FITC-labeled *Bifidobacterium* and intranasal instillation of MSNs-Bi (FITC labeling for *Bifidobacterium*)

Most studies on the gut–brain axis have focused on ascending signals from the intestine, with less attention on signals from the central nervous system [37, 38]. In addition, bacteria as nanoparticle carriers can enhance tissue penetration [31]. Therefore, to explore the biodistribution of MSNs-Bi after nasal instillation in mice, FITC-labeled MSNs-Bi were injected nasally into C57BL/6J mice. PBS and FITC-labeled *Bifidobacterium* served as controls. Tissue fluorescence imaging was performed 5 h after post-injection. The results showed strong FITC fluorescence signals in the tissues of the brain, spinal cord, and abdominal cavity of MSNs-Bi-treated mice, but weak fluorescence in the PBS and *Bifidobacterium-*treated mice (Fig. 3a). Furthermore, no signal was detected in the lungs of MSNs-Bi-treated mice (Fig. 3a; Additional file 1: Fig. S6), indicating that the MSNs-Bi signal in the intestine is unlikely to be the result of swallowing mucus, but rather a signal sent from the brain to the gut. Confocal imaging further revealed that a strong FITC signal could be detected in the brain, spinal cord, and abdominal cavity tissue slices, and this signal co-localized with Cy3 signals that labeled MSNs (Fig. 3b; Additional file 1: Fig. S5), suggesting that intranasal instilled MSNs-Bi could be transmitted from the brain to the gut. Overall, these results indicated that there is a long-distance transmission pathway from the brain to the gut for regulating the brain-gut connection.

Next, we further evaluated the viability of *Bifidobacterium* in the gut from MSNs-Bi after nasal administration. FITC-labeled *Bifidobacterium* and MSNs-Bi were administered orally and nasally to C57BL/6 J mice, respectively, and fresh organ contents (stomach, small intestine, and large intestine) were collected at 0, 0.25, 0.5, 3, 6, and 9 h for fluorescence imaging. Imaging results showed that a weak FITC fluorescence signal was detected in mice with oral *Bifidobacterium* only at 0.25 h post-injection (Fig. 3c). In contrast, in mice treated with nasal MSNs-Bi, there was a strong FITC fluorescence signal at 6 and 9 h post-injection, but no fluorescence at 0.25 h (Fig. 3c), again confirming that the signal in the gut is not the result of swallowing mucus. The results also indicated that MSNs could effectively improve the survival rate of *Bifidobacterium* in SIF. Altogether, these results suggest that nasal instillation of MSNs-Bi can significantly improve the survival of *Bifidobacteria* compared with conventional oral administration of *Bifidobacteria*.

### Nasal instillation of MSNs-Bi modulates intestinal microbiome

**Fig.4:**
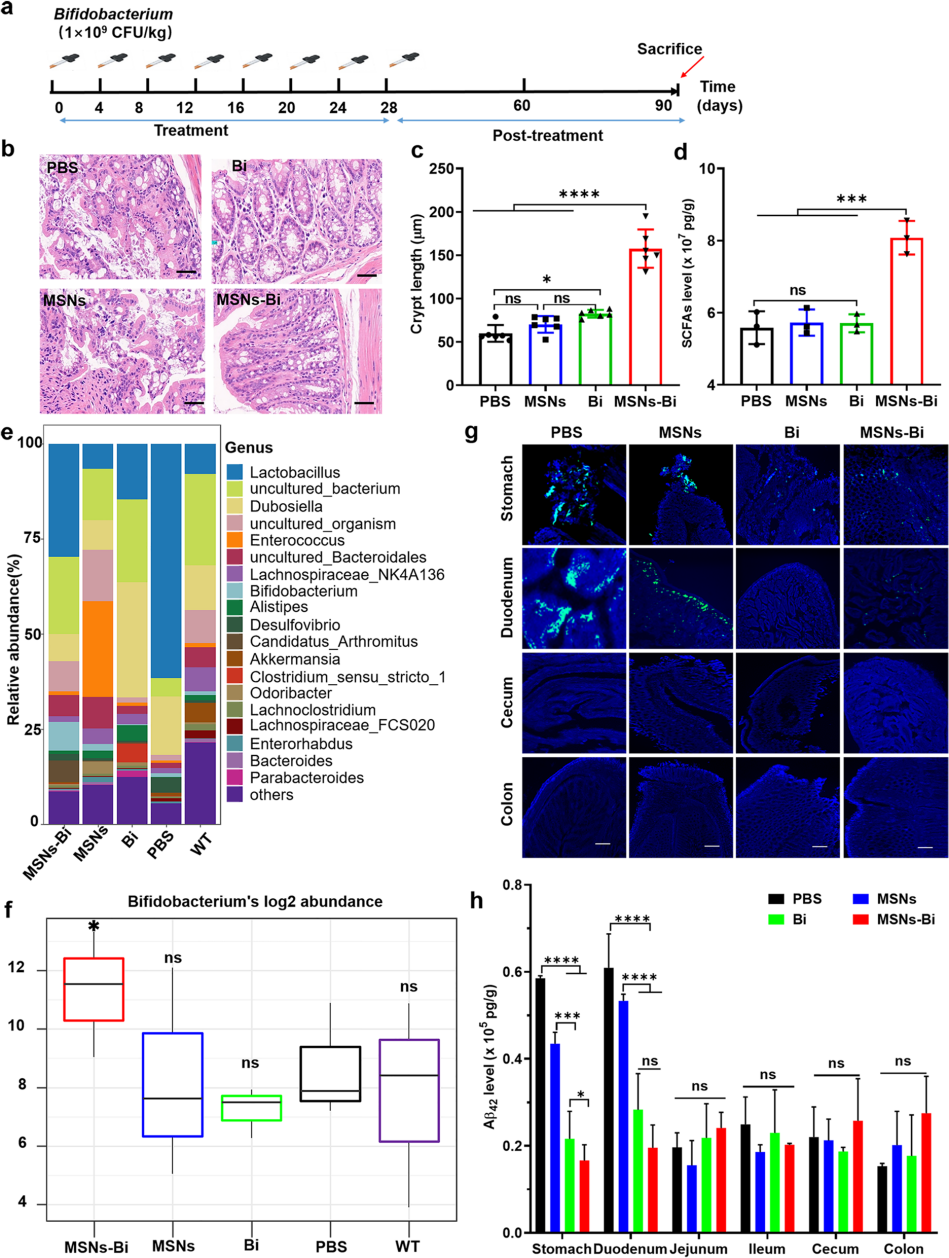
Nasal instillation of MSNs-Bi reduced intestinal inflammation and shaped gut microbiota. **a** Schematic diagram of MSNs-Bi treatment of 4 month-old APP/PS1 mice; n = 3. **b** Representative hematoxylin and eosin-stained images of colon tissues from APP/PS1 mice after treatment with PBS, MSNs, *Bifidobacterium*, and MSNs-Bi; Bi refers to *Bifidobacterium*, scale bars = 100 µm. **c** Crypt length of colon tissues from APP/PS1 mice treated with PBS, MSN, *Bifidobacterium*, and MSNs-Bi. **d** SCFAs levels in feces of APP/PS1 mice treated with PBS, MSNs, MSNs-Bi, and *Bifidobacterium*. **e** Composition of the gut microbiome of APP/PS1 mice treated with PBS, MSNs, *Bifidobacterium*, and MSNs-Bi. The Y-axis represents the relative abundance of genus-level gut microbial taxa. **f** *Bifidobacterium* abundance (log2 transformed) in the gut microbiome of C57BL/6J mice (WT) and APP/PS1 mice treated with PBS, MSNs, *Bifidobacterium*, and MSNs-Bi. **g** Representative images of ThioS staining of the gut of APP/PS1 mice after treatment with PBS, MSNs, *Bifidobacterium*, and MSNs-Bi; scale bars = 100 µm. **h** Aβ_42_ levels in the stomach, duodenum, jejunum, ileum, cecum, and colon of APP/PS1 mice treated with PBS, MSNs, *Bifidobacterium*, and MSNs-Bi

Studies have shown that gut inflammation is closely associated with the development of AD pathology [39], and probiotics can exert anti-inflammatory effects by enhancing innate immunity [40]. In our treatment study, 4 month-old APP/PS1 mice were intranasally administered MSNs-Bi, MSNs, or PBS every 4 days for a total of seven doses, and then allowed to recover for 8 weeks. Meanwhile, *Bifidobacterium* was orally gavaged as a control (Fig. 4a). H&E staining results of cross-sections of mouse colon tissue showed that compared with MSNs-Bi-treated mice, mice treated with PBS, MSNs, and *Bifidobacterium* had more severe colonic mucosal damage and longer colonic crypt lengths (*P* < 0.0001; Fig. 4b–c). Our previous study showed significant Aβ plaque enrichment in the gut of AD transgenic mice [41], leading to increased intestinal permeability and inflammation [42]. In addition, the effect of nasal delivery of PBS, MSNs, and MSNs-Bi on gut microbiota composition was investigated in 7 month-old APP/PS1 mice by sequencing bacterial 16 s rRNA genes. Sequencing of the V3–V4 region of the small intestinal contents showed a significantly higher abundance of *Bifidobacterium* (*P* = 0.032) in the MSNs-Bi treatment group compared with the other treatment groups, but not for the *Bifidobacterium*-treated group (Fig. 4e–f), while the α-diversity index (Chao1, ACE, Simpson, and Shannon based on OUT level) did not vary significantly among the four treatment groups (Additional file 1: Fig. S7). Compared with WT mice, PBS-treated AD mice showed no significant alterations in gut microbial composition (Fig. 4e–f). The above results suggested that while chronic intranasal instillation of MSNs-Bi increased the proportion of anti-inflammatory microbes in the gut microbiota and possibly reduced gut inflammation, it did not significantly increase or alter gut microbial abundance.

To further evaluate the effect of different treatments on intestinal soluble Aβ, tissue grinding and sectioning were performed on the intestines of the above-treated mice. Confocal imaging of intestinal tissue showed that the area fraction of ThioS-positive Aβ plaques in the stomach and duodenum of MSNs-Bi-treated mice was significantly lower than that of PBS- and MSNs-treated mice (*P* < 0.0001, Fig. 4g, Additional file 1: Fig. S8). Furthermore, IP-Western blotting results showed that Aβ bands were only present in the brain, spinal cord, blood, stomach, and duodenum of mice treated with PBS and MSNs (Additional file 1: Fig. S9). Quantitative results showed that Aβ levels in the stomach and duodenum of mice treated with *Bifidobacterium* and MSNs-Bi were significantly decreased compared with those of PBS and MSN-treated mice (*P* < 0.0001). Compared with other treatment groups, the MSNs-Bi treatment group had the lowest Aβ level in gastric tissue (Fig. 4h), a finding consistent with the results of ThioS-stained intestinal tissue fluorescence imaging. Interestingly, no significant changes in Aβ levels were observed in mouse jejunum, ileum, cecum, and colon between different treatment groups (Fig. 4g–h). The above results indicated that intranasally delivered MSNs-Bi can reduce Aβ deposition in the gastrointestinal tract.

Studies have shown that the probiotic metabolite SCFA can inhibit Aβ accumulation and alleviate intestinal inflammation [43]. To explore the relationship between intestinal inflammation and SCFAs, we assessed the content of SCFAs in feces. The results showed that the levels of SCFAs in the intestinal tissue of AD mice treated with MSNs-Bi were significantly increased compared with AD mice treated with PBS, *Bifidobacterium,* and MSNs (*P* < 0.001; Fig. 4d). The findings suggested that nasal delivery of MSNs-Bi can reduce intestinal inflammation and decrease intestinal Aβ content by increasing the SCFAs content.

### Nasal instillation of MSNs-Bi reduces Aβ deposition and neuroinflammation in the brain

**Fig.5:**
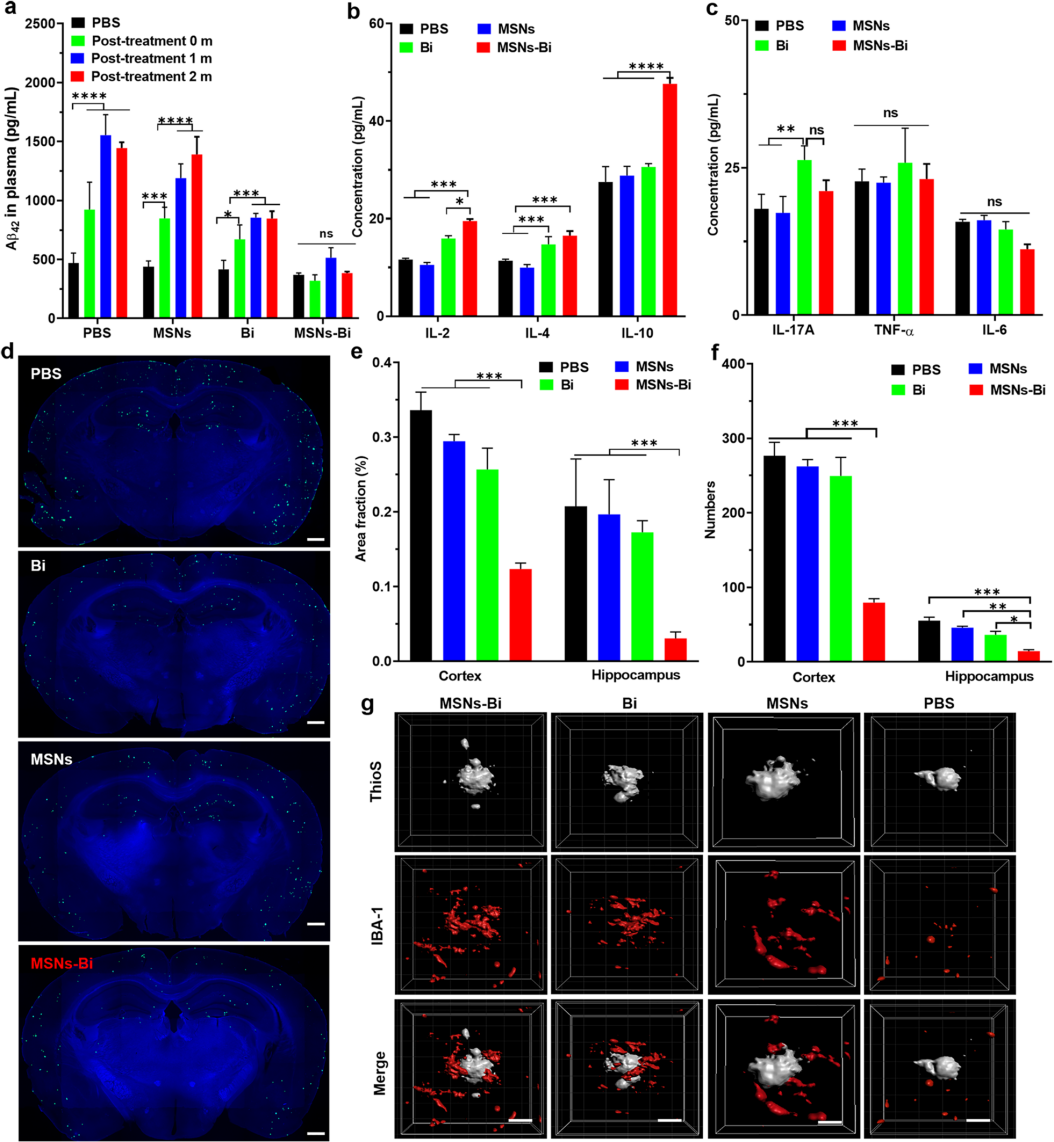
Nasal instillation of MSNs-Bi reduced Aβ deposition and neuroinflammation in APP/PS1 mice. **a** Changes in blood Aβ levels in APP/PS1 mice treated with PBS, MSNs, *Bifidobacterium*, and MSNs-Bi at 1, 2, and 3 months after the first treatment. Bi refers to *Bifidobacterium.* Expression levels of anti-inflammatory factors IL-2, IL-4, and IL-10 **b** and pro-inflammatory factors IL-17A, TNF-α, and IL-6 (**c)** in the blood of APP/PS1 mice treated with PBS, MSNs, *Bifidobacterium*, and MSNs-Bi for 1 month. **d** Representative confocal images of APP/PS1 mouse brain sections treated with PBS, MSNs, *Bifidobacterium*, and MSNs-Bi; scale bar = 500 µm. **e, f** Percentage area fraction and the number of ThioS-positive Aβ plaques in the hippocampus and cortex of APP/PS1 mice treated with PBS, MSNs, *Bifidobacterium*, and MSNs-Bi for 1 month. **g** Representative confocal images of three-dimensional reconstruction of the cortex, depicting Aβ (grey) phagocytosed by IBA-1^+^ microglia (red); scale bar = 30 µm; Bi refers to *Bifidobacterium.* Data are presented as mean ± SD. Two-way ANOVA, *p < 0.05, **p < 0.01, ***p < 0.001

We investigated whether intranasal instillation of MSNs-Bi could effectively reduce brain Aβ levels. Aβ levels in the peripheral blood of mice treated with PBS, *Bifidobacterium*, MSNs, and MSNs-Bi were significantly increased at 1, 2, and 3 m after the first treatment compared with pre-treatment levels (*P* < 0.001; Fig. 5a). In addition, the detection results of inflammatory factors showed that the levels of pro-inflammatory factors interleukin 2 (IL-2), interleukin 10 (IL-10), and interleukin-4 (IL-4) in the mice in the MSNs-Bi treatment group were significantly increased compared with the mice in the PBS and MSNs treatment groups (*P* < 0.0001, Fig. 5b). In addition, the anti-inflammatory factor IL-10 in the blood of MSNs-Bi-treated mice was 1.7-fold higher than that of *Bifidobacterium*-treated mice (Fig. 5b). However, the levels of pro-inflammatory factors including tumor necrosis factor-α (TNF-α) and interleukin-6 (IL-6) did not change significantly between treatment groups (Fig. 5c).

To investigate the effect of intranasal instillation of MSNs-Bi on the number of Aβ plaques in the brain, ThioS staining was performed on brain sections of APP/PS1 mice in different treatment groups. Confocal fluorescence imaging revealed significantly fewer ThioS-positive Aβ plaques in the cortex (*P* < 0.0001) and HIP (*P* < 0.0001; Fig. 5d–e) of MSNs-Bi-treated mice compared with other treated mice. Furthermore, ThioS-positive Aβ plaques in the cortex of *Bifidobacterium*-treated mice were significantly reduced (*P* = 0.0038), but not the HIP (*P* = 0.7602), compared with PBS-treated mice (Fig. 5e), suggesting that oral instillation of *Bifidobacterium* attenuated AD disease progression, which is consistent with previous reports [44]. However, MSNs-Bi more significantly reduced the number of Aβ plaques in mouse cortex compared with oral administration of *Bifidobacterium* (*P* < 0.0001; Fig. 5f), suggesting that nasal instillation of MSNs-Bi alleviated brain Aβ burden of APP/PS1 mice, and the effect is better than oral administration of *Bifidobacterium*.

Aβ plaques can strongly activate microglia to produce pro-inflammatory cytokines that promote the pathological process of AD [15, 45]. Therefore, we further evaluated the effect of nasal delivery of MSNs-Bi on neuroinflammation. Immunofluorescence results showed that compared with mice treated with PBS and MSNs, the cerebral cortex of mice treated with MSNs-Bi and *Bifidobacterium* had denser microglial protrusions, with larger diameters of microglial cell bodies in MSNs-Bi-treated mice (Fig. 5g). These findings suggest that intranasal instillation of MSNs-Bi can inhibit the activation of microglia, which have anti-inflammatory effects during AD treatment.

### Nasal instillation of MSNs-Bi improves olfactory dysfunction in APP/PS1 mice

**Fig.6:**
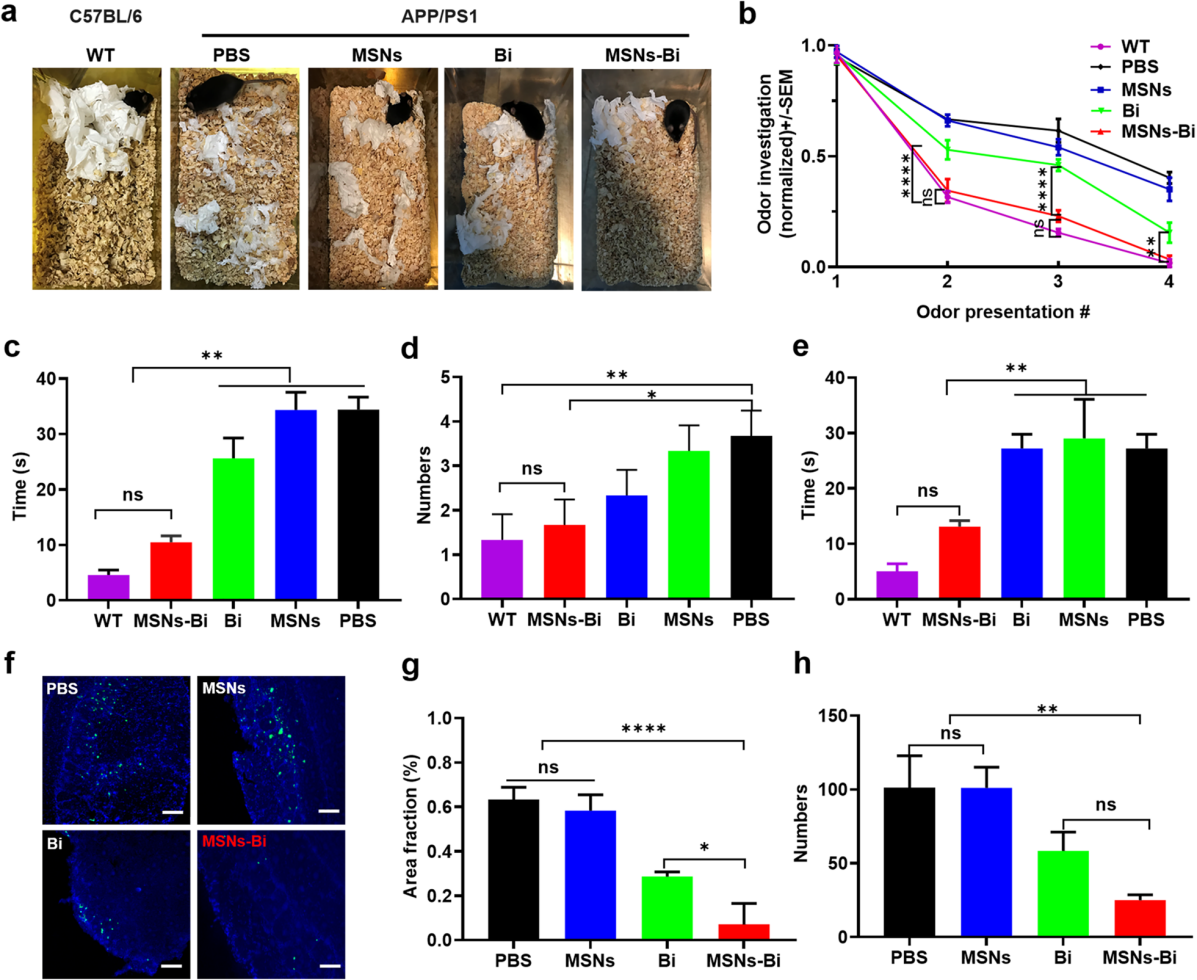
Nasal instillation of MSNs-Bi ameliorated olfactory dysfunction in APP/PS1 mice. **a** Comparison of nesting behavior in C57BL/6 mice (WT) and age-matched APP/PS1 mice treated with PBS, MSNs, *Bifidobacterium*, and MSNs-Bi. **b** Odor habituation (normalized) in four consecutive odor presentation trials in WT mice and age-matched APP/PS1 mice treated with PBS, MSNs, *Bifidobacterium*, and MSNs-Bi. (n = 3 mice per group; two-tailed t-test; mean ± SEM, **P < 0.01, ***P < 0.001). Time **c,** numbers **d**, and the total time of penalty points **e** within 300 s of first reaching the safe platform. **f** Representative confocal images of ThioS-stained Aβ plaques in the olfactory bulbs in APP/PS1 mice after PBS, MSNs, and *Bifidobacterium* treatment for 1 month. The number **g** and percentage area fraction **h** of ThioS-positive Aβ plaques in the olfactory bulb tissues of APP/PS1 mice treated with PBS, MSNs, *Bifidobacterium*, MSNs-Bi. (scale bars = 100 µm; Bi refers to *Bifidobacterium*)*.* Two-way ANOVA, *P < 0.05, **P < 0.01, ***P < 0.001

Social nesting is a typical species behavior that occurs naturally under standard housing conditions and can be a useful tool for assessing animal welfare, monitoring disease progression, and evaluating the effectiveness of prevention/treatment strategies. Our results showed that APP/PS1 mice exhibited lower social nesting activity compared to WT mice (C57BL/6J), which is consistent with previous reports [46]. Our findings further showed that nesting behavior was disrupted in APP/PS1 mice treated with PBS, MSNs, and *Bifidobacterium*, but restoration of impaired nesting behavior was only observed in MSNs-Bi-treated APP/PS1 mice (Fig. 6a). It is well known that anosmia is one of the earliest symptoms of AD [47]. A correlation between olfactory dysfunction and Aβ has been reported in APP/PS1 mice [48]. Therefore, we evaluated the repair effect of nasal delivery of MSNs-Bi on olfactory dysfunction in APP/PS1 mice. The results of the odor cross-habituation test showed that MSNs-Bi-treated APP/PS1 mice spent significantly less time searching for odors 2 (*P* < 0.0001), 3 (*P* < 0.0001), 4 (*P* = 0.001) than *Bifidobacterium*-treated mice, indicating that the odor habituation behavior of APP/PS1 mice was significantly improved after MSNs-Bi treatment (Fig. 6b). Notably, MSNs-Bi-treated APP/PS1 mice and WT mice showed no significant difference in the performance of all odors (Fig. 6b). Therefore, nasally administered MSNs-Bi can greatly improve the olfactory impairment in APP/PS1 mice.

Next, we evaluated the effect of nasally delivered MSNs-Bi on the improvement in cognitive dysfunction in APP/PS1 mice. In the step-down test, MSNs-Bi-treated and WT mice found a safe plateau more quickly when they received the first electrical stimulation compared with PBS, MSNs, and *Bifidobacterium*-treated mice (*P* < 0.002, Fig. 6c). After four training sessions, the mice were tested for memory and cognitive abilities within 300 s. The results showed that MSNs-Bi-treated mice received significantly fewer punishments (*P* = 0.0171; Fig. 6d) compared with PBS-treated mice. In addition, the total electrical shock duration was significantly shorter in MSNs-Bi-treated mice compared with PBS, MSNs- and *Bifidobacterium*-treated mice (*P* = 0.0078; Fig. 6e). The results of ThioS staining in olfactory bulbs of APP/PS1 mice showed that MSNs-Bi-treated mice showed significantly reduced ThioS-positive Aβ plaques in olfactory bulbs compared with mice in other treatment groups (Fig. 6f).

Next, the area and number of Aβ plaques were quantified by stereological analysis. The results showed that the area fraction (*P* < 0.0001; Fig. 6g) and number (*P* < 0.0012; Fig. 6h) of ThioS-positive Aβ plaques in olfactory bulbs of mice treated with *Bifidobacterium* and MSNs-Bi were significantly lower than those in PBS- and MSNs-treated mice. In addition, the area fraction of ThioS-positive Aβ plaques in the olfactory bulbs of MSNs-Bi-treated mice was significantly lower than that in olfactory bulbs of *Bifidobacterium*-treated mice (*P* = 0.0236; Fig. 6g), demonstrating that nasally delivered MSNs-Bi could inhibit the formation of Aβ plaques in olfactory bulbs. Altogether, our findings suggest that nasally delivered MSNs-Bi improves olfactory behavior in AD mice.

## Discussion

In this study, we reported the efficacy of long-distance nasal delivery of MSN-loaded *Bifidobacterium* to the gut of AD transgenic mice in reducing intestinal inflammation and brain Aβ burden. We found that intranasal instillation of MSNs-Bi could propagate to the brain and gastrointestinal tract. Interestingly, encapsulation of *Bifidobacterium* with MSNs could effectively prevent the problem of easy inactivation of *Bifidobacterium* through the gastrointestinal environment. Notably, during the treatment of 4 month-old APP/PS1 mice, we found that intranasal instillation of MSNs-Bi was effective in reducing intestinal inflammation, increasing the percentage of *Bifidobacterium* in the gut microbiota and intestinal SCFA levels. In addition, Aβ levels in the brain and small intestine were significantly reduced, and neuroinflammation and cognitive behavior were significantly improved. All results suggested that nasal delivery of MSNs-Bi could improve intestinal inflammation and AD pathology.

The inflammatory infection hypothesis of AD suggests that AD pathogenesis may begin in the gut and is closely related to gut microbiota-driven inflammation [49]. Intestinal dysbiosis increases the abundance of pro-inflammatory bacterial species [50] that produce amyloids, LPS, and other immunogenic compounds to promote intestinal inflammation [51, 52], which in turn activate C/EBPb/d-secretase to initiate the propagation of Aβ and Tau in AD [37, 53]. Studies have shown that increasing the abundance of probiotics such as *Bifidobacterium* in the intestinal flora can effectively increase the production of SCFAs [54]. SCFAs are the main metabolites of the intestinal flora, which have immunomodulatory functions and can reduce intestinal inflammation. In our study, nasally delivered MSNs-Bi significantly increased *Bifidobacterium* proportion in the gut microbiota and SCFAs in feces of APP/PS1 mice, and reduced gut inflammation, which in turn attenuated brain neuroinflammation and Aβ burden (Figs. 4, 5). This may be because intestinal SCFAs can cross the BBB [55] and reduce neuroinflammation and Aβ accumulation in the brain by inhibiting the activation of microglia and astrocytes.

To date, although oral probiotics can reduce the pathology of Aβ and tau in AD animal models, gastric acid kills microorganisms to a large extent [56]. In our study, MSNs-Bi exhibited excellent stability over 12 h and well maintained the vitality of *Bifidobacterium* in SIF, which was extremely important for their successful delivery (Fig. 1d–e**; **Fig. 2c–d). Nasal delivery is a direct route of administration for the treatment of central nervous system disorders. Although the olfactory circuit has been elucidated, the route of drug delivery in the brain remains unclear [48]. After intranasal instillation of MSNs-Bi, both *Bifidobacterium*-labeled FITC and MSNs-labeled Cy3 signals could be observed in both the abdominal cavity and the gut (Fig. 3a–b), suggesting a potential peripherally derived long-distance transmission pathway from the brain to the gut. Notably, nasally delivered MSNs-Bi increased the percentage of *Bifidobacterium* in the gut microbiota of APP/PS1 mice and reduced intestinal inflammation (Fig. 4b–f). Therefore, intranasal delivery of MSNs-encapsulated probiotics or small chemical molecules may be an effective means for anti-inflammatory therapy in the gastrointestinal tract.

Since Aβ deposition can lead to neuronal atrophy, dendritic abnormalities, synaptic loss, and axonal degeneration, several studies have shown a correlation between olfactory dysfunction and Aβ deposition [57]. Aβ has been shown to exhibit spatiotemporal deposition in AD mice, and Aβ deposition sequentially extends to the olfactory epithelium (1–2 months), OB (3–4 months), HIP (6–7 months), and central cortex (9–10 months) [48]. The entorhinal cortex can preprocess information entering the hippocampus and facilitate learning and memory, while Aβ deposits in the entorhinal cortex-hippocampus connection [58]. Therefore, cognitive deficits are associated with the degree of olfactory dysfunction. In this study, we found that intranasally delivered MSNs-Bi effectively reduced the accumulation of Aβ aggregates in the OB and HIP, and also ameliorated olfactory dysfunction (Fig. 6). Olfactory dysfunction occurs in the early stages of AD; therefore, we speculate that MSNs-Bi attenuates Aβ pathology by clearing Aβ accumulation in the olfactory bulb at 3 months and reduced Aβ deposition in the hippocampus at 7 months.

In summary, with the help of MSNs, the long-distance transport of *Bifidobacterium* from the nasal cavity to the intestine can be achieved, breaking the traditional delivery method of probiotics and the long-distance limitation of nasal delivery. In APP/PS1 mice, intranasally delivered MSNs-Bi increased *Bifidobacterium* abundance and SCFA levels, reduced intestinal inflammation and brain Aβ burden, and improved olfactory impairment. Furthermore, our study demonstrated that modulating gut microbiota and reducing gut inflammation can improve cognitive impairment in AD. Thus, our study provides a new avenue for drug delivery in intestinal diseases and a new perspective for AD treatment.

### Supplementary Information

**Additional file 1: Figure S1.** Characterization of MSNs.** a** Transmission electron micrographs of MSNs of different sizes (scale bar = 200 nm). **b **Corresponding histogram and Gaussian fit of the measured MSN size distribution. **Figure S2.** Absorption spectroscopy of MSN-Cy3, FITC-*Bifidobacterium*, and MSNs-*Bifidobacterium*. **Figure S3.** Representative images of colonies formed by MSNs-Bi on the culture medium were used to evaluate the activity of *Bifidobacterium* in Krebs-Henseleit solution. **Figure S4.** Characterization of MSN-encapsulated *Bifidobacterium *and* E. coli****. ***MSNs loading rates for *Bifidobacterium* (**a**) and *E. coli* (**b**) and the viability of *Bifidobacterium* (**c**) and *E. coli* (**d**) during exposure to SIF. **Figure S5.** Distribution of MSNs-Bi in the gastrointestinal tract after intranasal administration. DAPI (blue), FITC (green), and Cy3 (Red). (scale bar = 200 μm). **Figure S6.** Distribution of MSNs-Bi in the lung after intranasal administration. DAPI (blue), FITC (green), and Cy3 (Red). (scale bar = 200 μm). **Figure S7.** Alpha diversity analysis of the gut microbiome of C57BL/6 mice (WT) and APP/PS1 mice treated with PBS, MSNs, *Bifidobacterium*, and MSNs-Bi. Bi refers to *Bifidobacterium*. Boxplots show the index of Chao1, ACE, and Shannon. **Figure S8.** The area fraction of ThioS-stained Aβ plaques in the stomach, duodenum, jejunum, ileum, cecum, and colon from APP/PS1 mice treated with PBS, MSNs, *Bifidobacterium*, and MSNs-Bi. Two-way ANOVA, ****P < 0.001. **Figure S9.** IP-Western blotting images of the brain, spinal cord, blood, stomach, duodenum, jejunum, ileum, cecum, and colon from APP/PS1 mice treated with PBS, MSNs, *Bifidobacterium*, and MSNs-Bi. **Figure S10.** Relative fluorescence intensity was used to monitor changes in the level of Cy3 in MSNs.
