# Correction: Mesoporous silica nanoparticle-encapsulated *Bifidobacterium* attenuates brain Aβ burden and improves olfactory dysfunction of APP/PS1 mice by nasal delivery

**DOI:** 10.1186/s12951-024-02395-7

**Published:** 2024-04-06

**Authors:** Ni Liu, Changwen Yang, Xiaohan Liang, Kai Cao, Jun Xie, Qingming Luo, Haiming Luo

**Affiliations:** 1 Britton Chance Center for Biomedical Photonics, Wuhan National Laboratory for Optoelectronics, Huazhong University of Science and Technology, WuhanHubei, 430074 China; 2 MoE Key Laboratory for Biomedical Photonics, School of Engineering Sciences, Huazhong University of Science and Technology, Wuhan, China; 3 School of Biomedical Engineering, Hainan University, Haikou, 570228 Hainan China

**Correction: Journal of Nanobiotechnology (2022) 20:439** 10.1186/s12951-022-01642-z

Following publication of the original article [1], the authors identified an error in Fig. 2 a and fig. 2c. This is to correct the images of Fig. 2a Bi + 10 µg/mL MSNs, Fig. 2c Bi + 40 µg/mL MSNs, and Fig. 2c MSNs-Bi + SIF 120 min.

The similar images of Fig. 2a Bi + 10 µg/mL MSNs, Fig. 2a Bi + 40 µg/mL MSNs, and Fig. 2c MSNs-Bi + SIF 120 min are caused by the change in image order when the images are transferred from the mobile phone to the computer. The correct figures are given below.

**Fig. 2:**
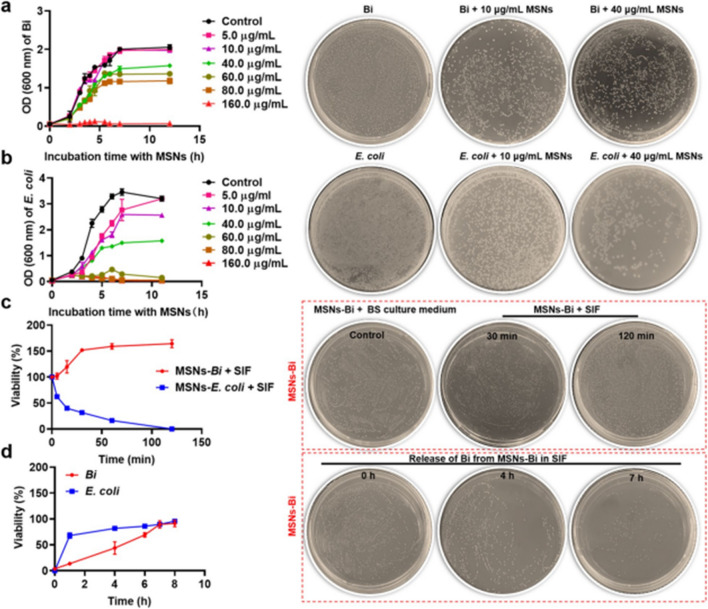
Effects of MSNs on the growth of *Bifidobacterium*. Changes in bacterial growth of **a** *Bifidobacterium* and **b** *Escherichia coli* (*E. coli*) in the presence of different MSN concentrations. **c** Viability of encapsulated *Bifidobacterium* and *E. coli* during exposure to simulated intestinal fluid (SIF) with bile salts. **d** Release of *Bifidobacterium* and *E. coli* from MSNs encapsulation in SIF at 37 °C. Viability represents the percentage of bacteria surviving relative to the initial population. Data are presented as mean ± SD, n = 3

Corrected Fig. 2a
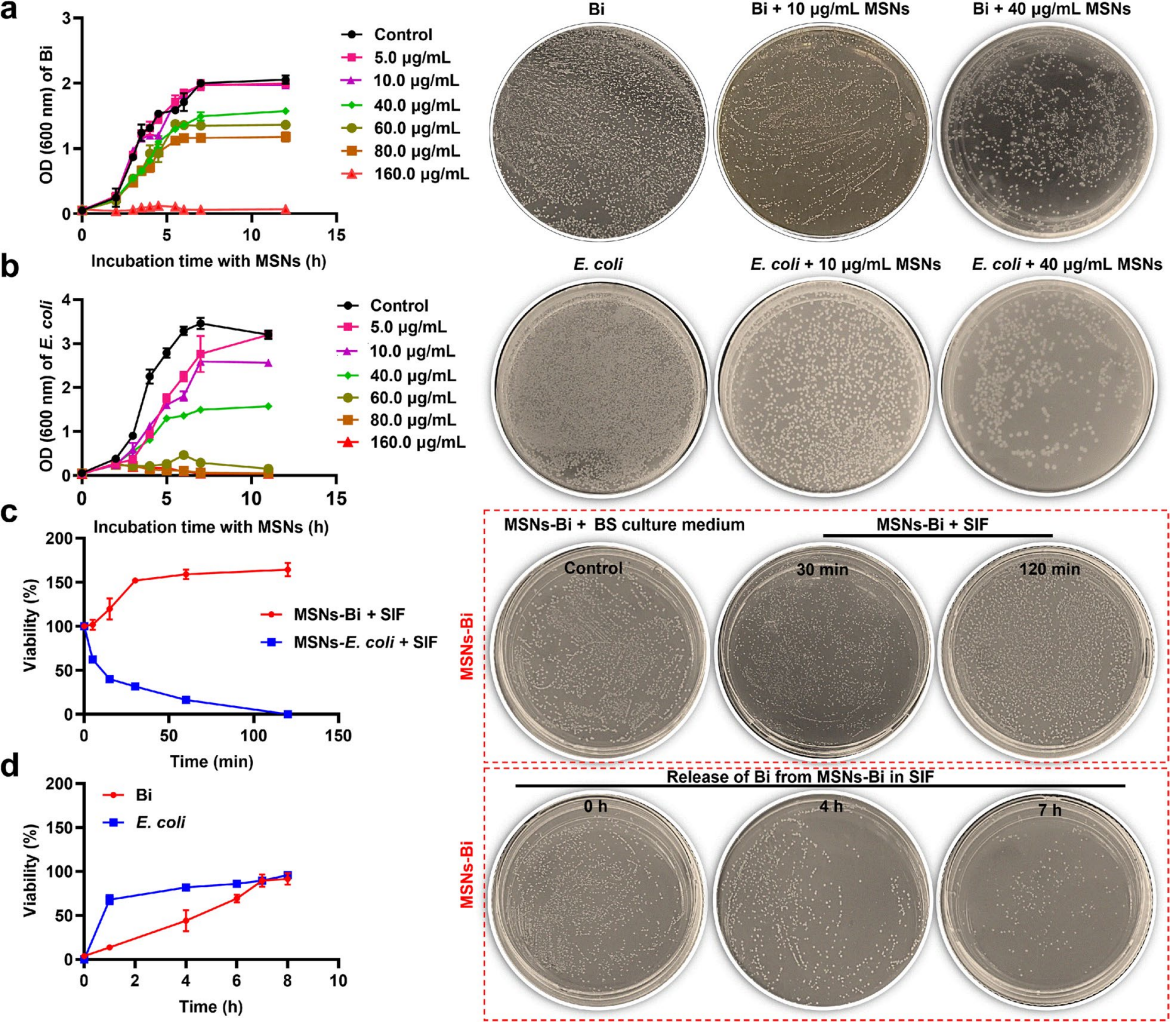


Incorrect Fig. 2a


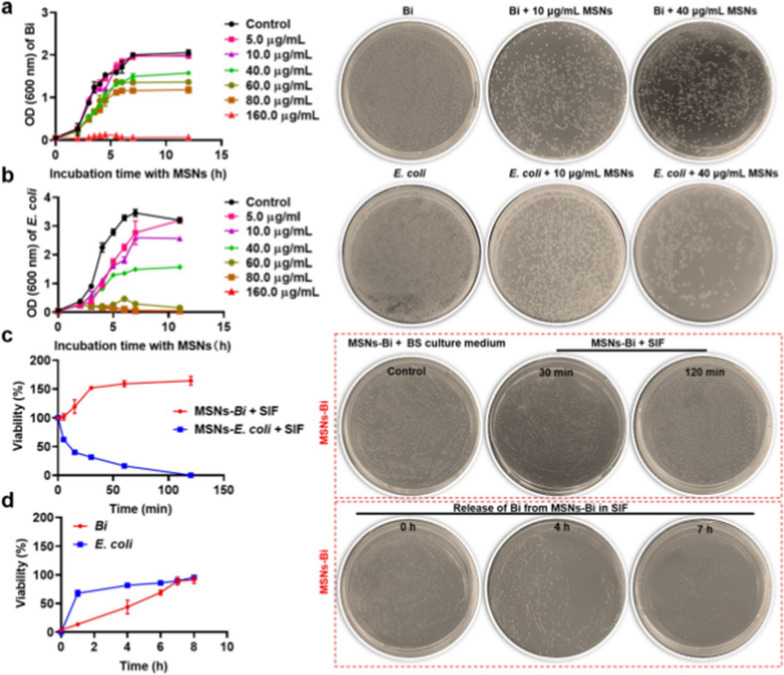


The original article [1] has been corrected.
