# Supplementary material for: Mesoporous silica nanoparticle-encapsulated Bifidobacterium attenuates brain Aβ burden and improves olfactory dysfunction of APP/PS1 mice by nasal delivery
Source: J Nanobiotechnology. 2022 Oct 7;20:439. doi: 10.1186/s12951-022-01642-z (PMC9547428; doi:10.1186/s12951-022-01642-z)
Supplement: Supplementary file 1 — Additional file 1: Figure S1. Characterization of MSNs. a Transmission electron micrographs of MSNs of different sizes (scale bar = 200 nm). b Corresponding histogram and Gaussian fit of the measured MSN size distribution. Figure S2. Absorption spectroscopy of MSN-Cy3, FITC-Bifidobacterium, and MSNs-Bifidobacterium. Figure S3. Representative images of colonies formed by MSNs-Bi on the culture medium were used to evaluate the activity of Bifidobacterium in Krebs-Henseleit solution. Figure S4. Characterization of MSN-encapsulated Bifidobacterium and E. coli. MSNs loading rates for Bifidobacterium (a) and E. coli (b) and the viability of Bifidobacterium (c) and E. coli (d) during exposure to SIF. Figure S5. Distribution of MSNs-Bi in the gastrointestinal tract after intranasal administration. DAPI (blue), FITC (green), and Cy3 (Red). (scale bar = 200 μm). Figure S6. Distribution of MSNs-Bi in the lung after intranasal administration. DAPI (blue), FITC (green), and Cy3 (Red). (scale bar = 200 μm). Figure S7. Alpha diversity analysis of the gut microbiome of C57BL/6 mice (WT) and APP/PS1 mice treated with PBS, MSNs, Bifidobacterium, and MSNs-Bi. Bi refers to Bifidobacterium. Boxplots show the index of Chao1, ACE, and Shannon. Figure S8. The area fraction of ThioS-stained Aβ plaques in the stomach, duodenum, jejunum, ileum, cecum, and colon from APP/PS1 mice treated with PBS, MSNs, Bifidobacterium, and MSNs-Bi. Two-way ANOVA, ****P < 0.001. Figure S9. IP-Western blotting images of the brain, spinal cord, blood, stomach, duodenum, jejunum, ileum, cecum, and colon from APP/PS1 mice treated with PBS, MSNs, Bifidobacterium, and MSNs-Bi. Figure S10. Relative fluorescence intensity was used to monitor changes in the level of Cy3 in MSNs. [file 12951_2022_1642_MOESM1_ESM.docx]

**Additional file 1 informations** for

Mesoporous silica nanoparticle-encapsulated *Bifidobacterium* attenuates brain Aβ burden and improves olfactory dysfunction in APP/PS1 mice by nasal delivery

Ni Liu^1,2^, Changwen Yang^1,2^, Xiaohan Liang^1,2^, Kai Cao^1,2^, Jun Xie^1,2^, Qingming Luo^1,2,3^, Haiming Luo^1,2^*****

*Correspondence: Haiming Luo, [hemluo@hust.edu.cn](mailto:hemluo@hust.edu.cn)；

**This PDF file includes:**

Supporting Information

Figs. S1 to S10

**Additional files information**

**
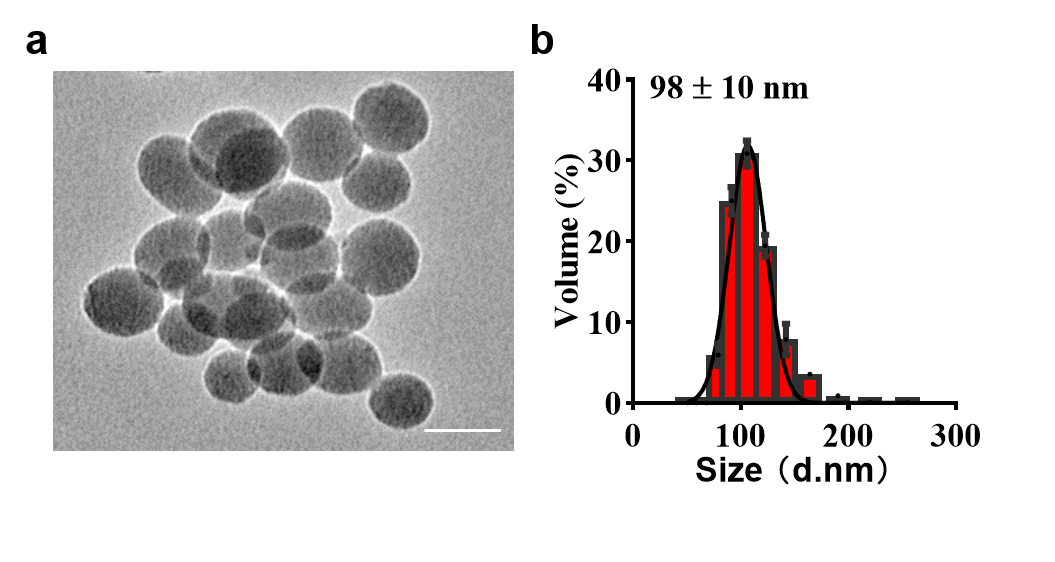
**

**Additional file 1: Figure S1** Characterization of MSNs. **a** Transmission electron micrographs of MSNs of different sizes (scale bar = 200 nm). **b** Corresponding histogram and Gaussian fit of the measured MSN size distribution.

**
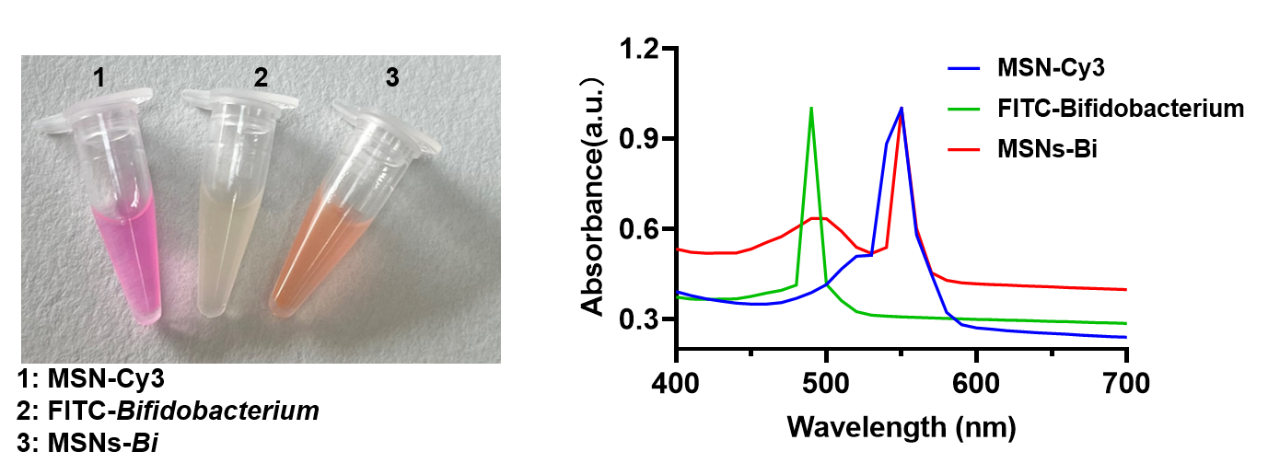
**

**Additional file 1: Figure S2** Absorption spectroscopy of MSN-Cy3, FITC-*Bifidobacterium*, and MSNs-*Bifidobacterium*.

**
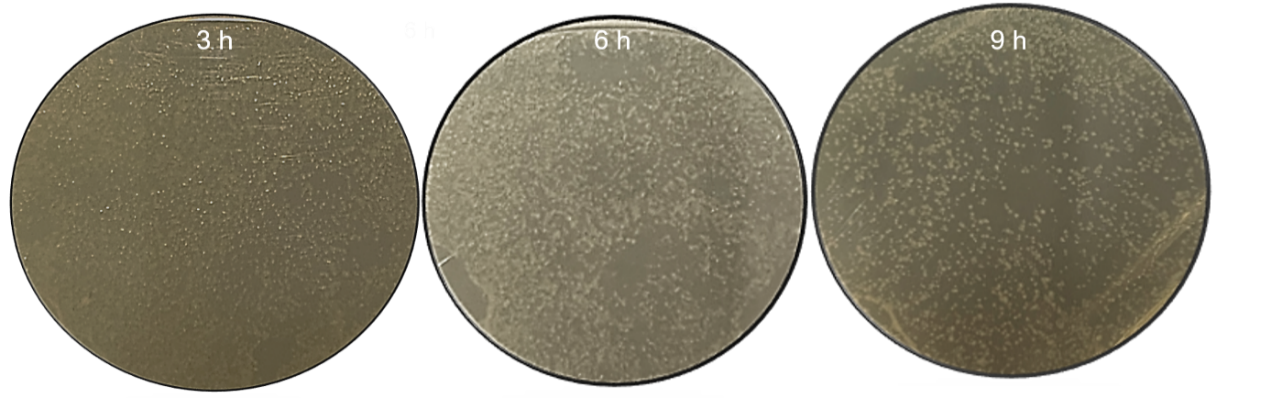
**

**Additional file 1: Figure S3** Representative images of colonies formed by MSNs-Bi on the culture medium were used to evaluate the activity of *Bifidobacterium* in Krebs-Henseleit solution.

**
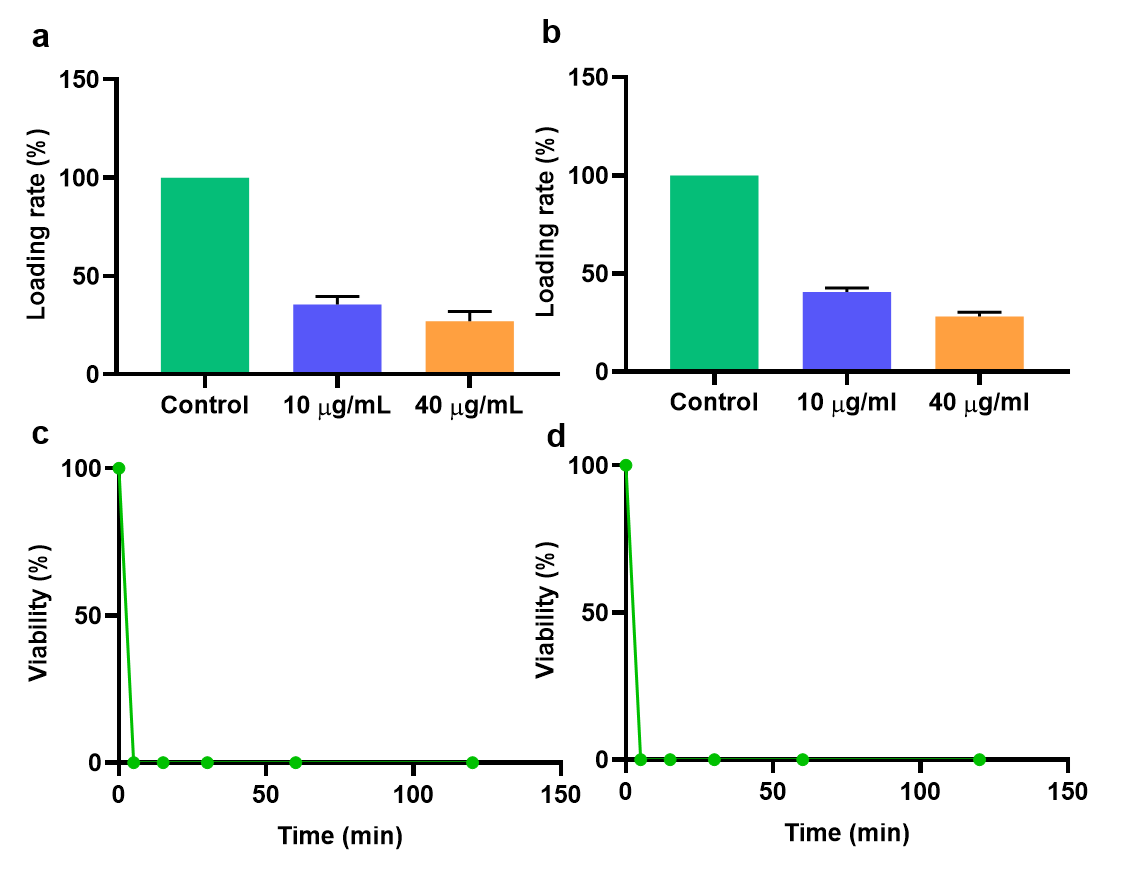
**

**Additional file 1: Figure S4** Characterization of MSN-encapsulated *Bifidobacterium* and *E. coli****.*** MSNs loading rates for *Bifidobacterium* (**a)** and *E. coli* (**b)** and the viability of *Bifidobacterium* (**c)** and *E. coli* (**d)** during exposure to SIF.


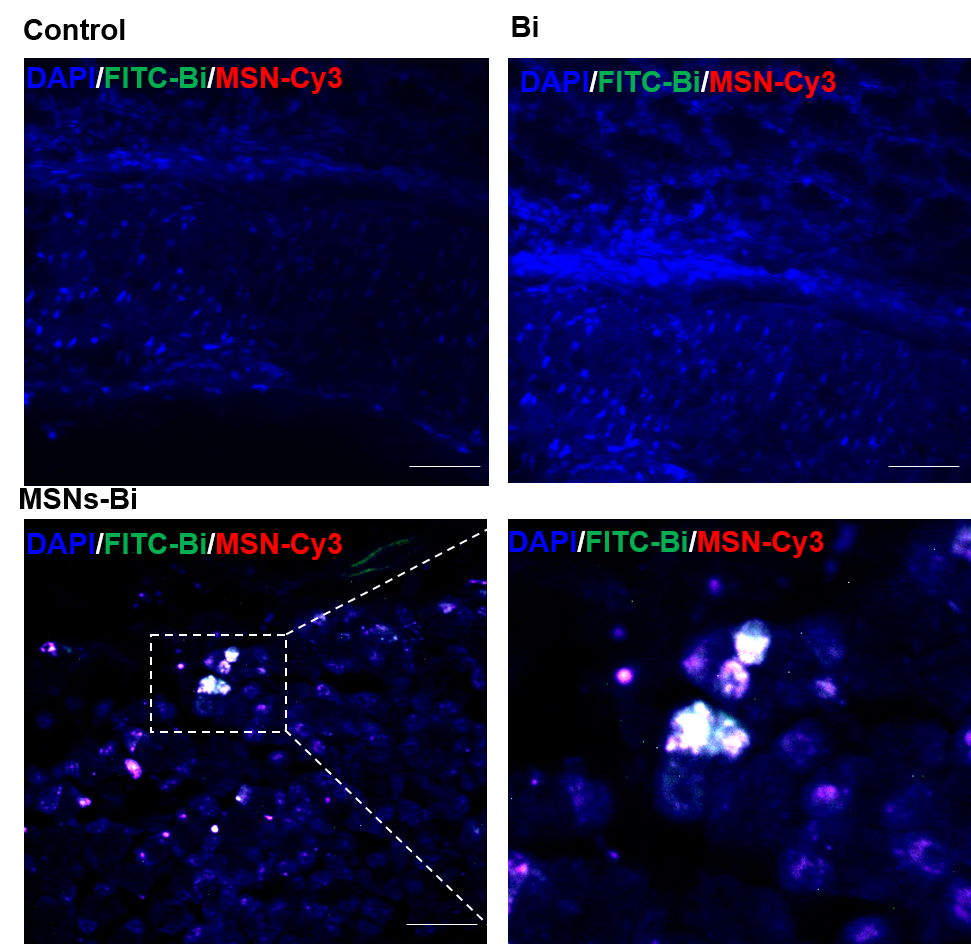


**Additional file 1: Figure S5** Distribution of MSNs-Bi in the gastrointestinal tract after intranasal administration. DAPI (blue), FITC (green), and Cy3 (Red). (scale bar = 200 μm).


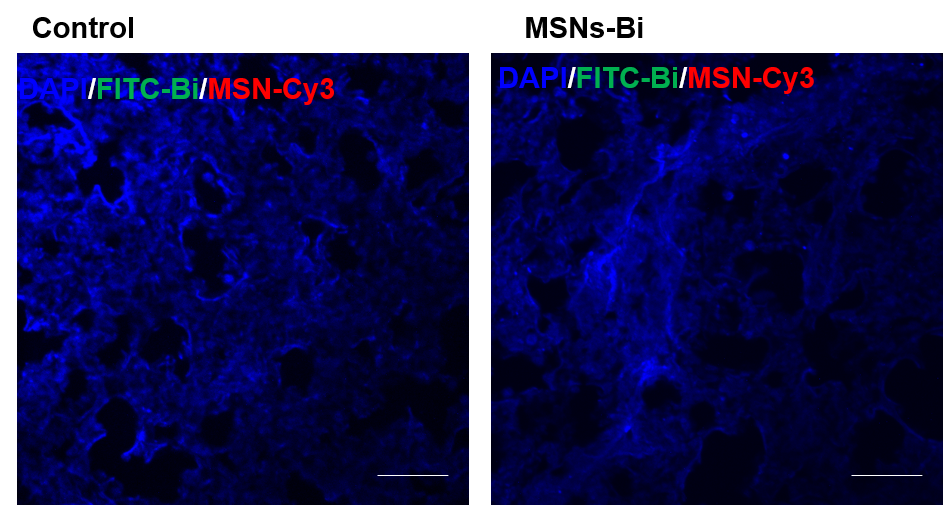


**Additional file 1: Figure S6** Distribution of MSNs-Bi in the lung after intranasal administration. DAPI (blue), FITC (green), and Cy3 (Red). (scale bar = 200 μm).


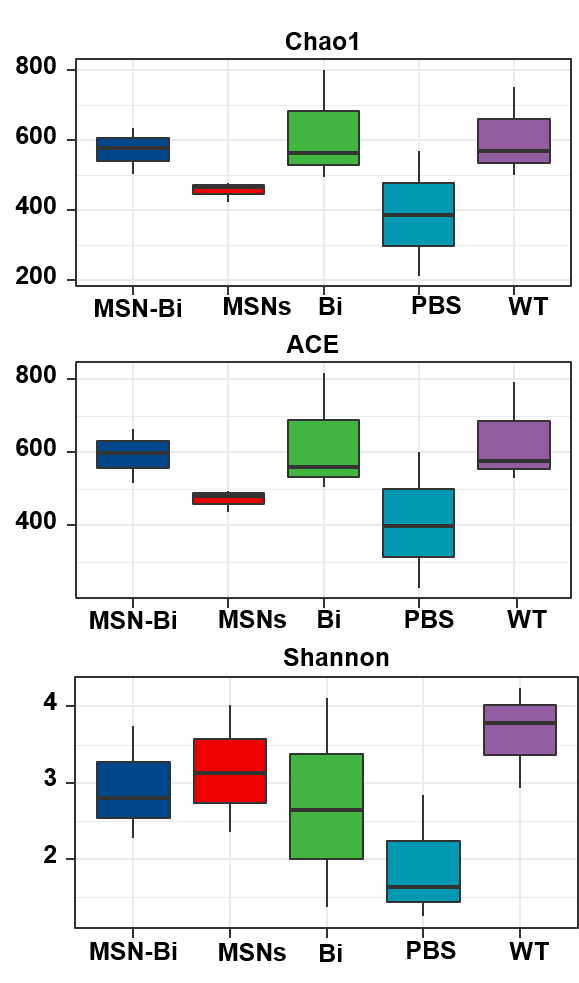


**Additional file 1: Figure S7** Alpha diversity analysis of the gut microbiome of C57BL/6 mice (WT) and APP/PS1 mice treated with PBS, MSNs, *Bifidobacterium*, and MSNs-Bi. Bi refers to *Bifidobacterium*. Boxplots show the index of Chao1, ACE, and Shannon.


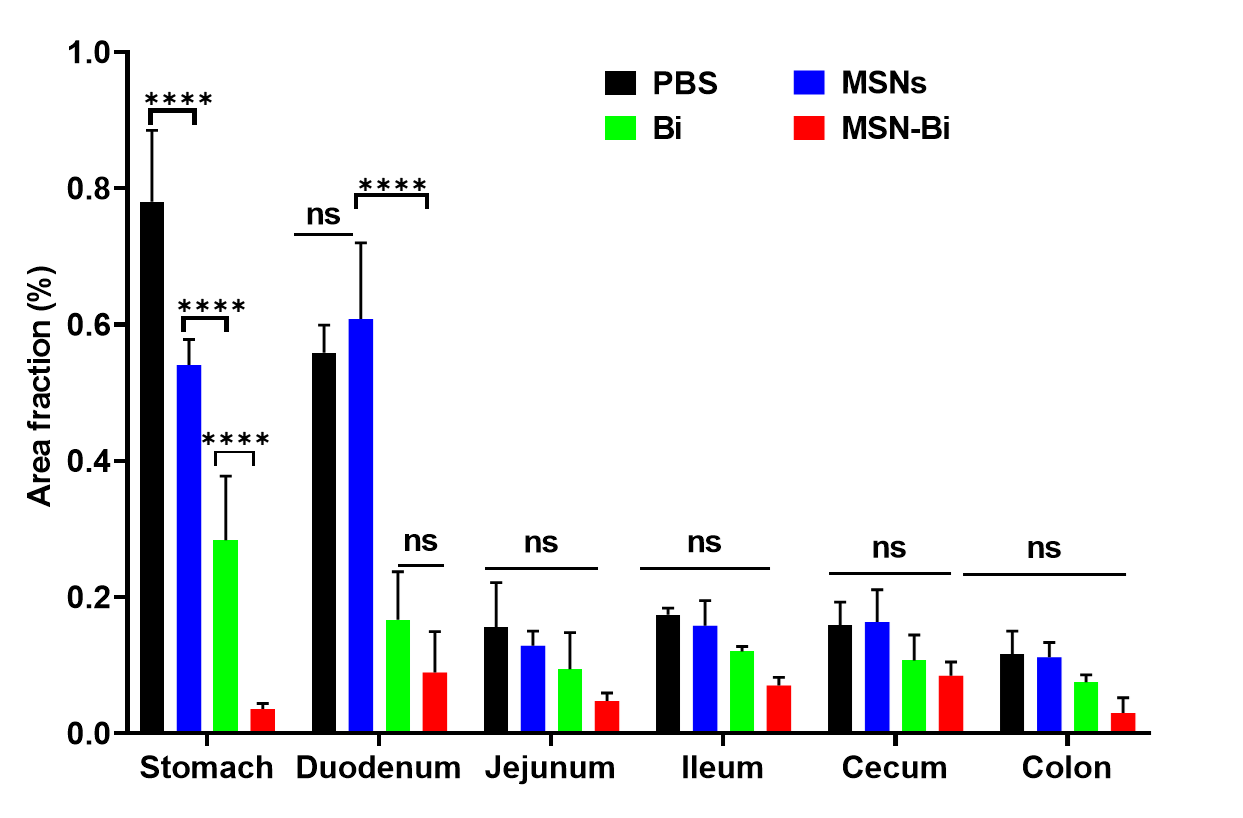


**Additional file 1: Figure S8** The area fraction of ThioS-stained Aβ plaques in the stomach, duodenum, jejunum, ileum, cecum, and colon from APP/PS1 mice treated with PBS, MSNs, *Bifidobacterium*, and MSNs-Bi. Two-way ANOVA, *****P* < 0.001.

**
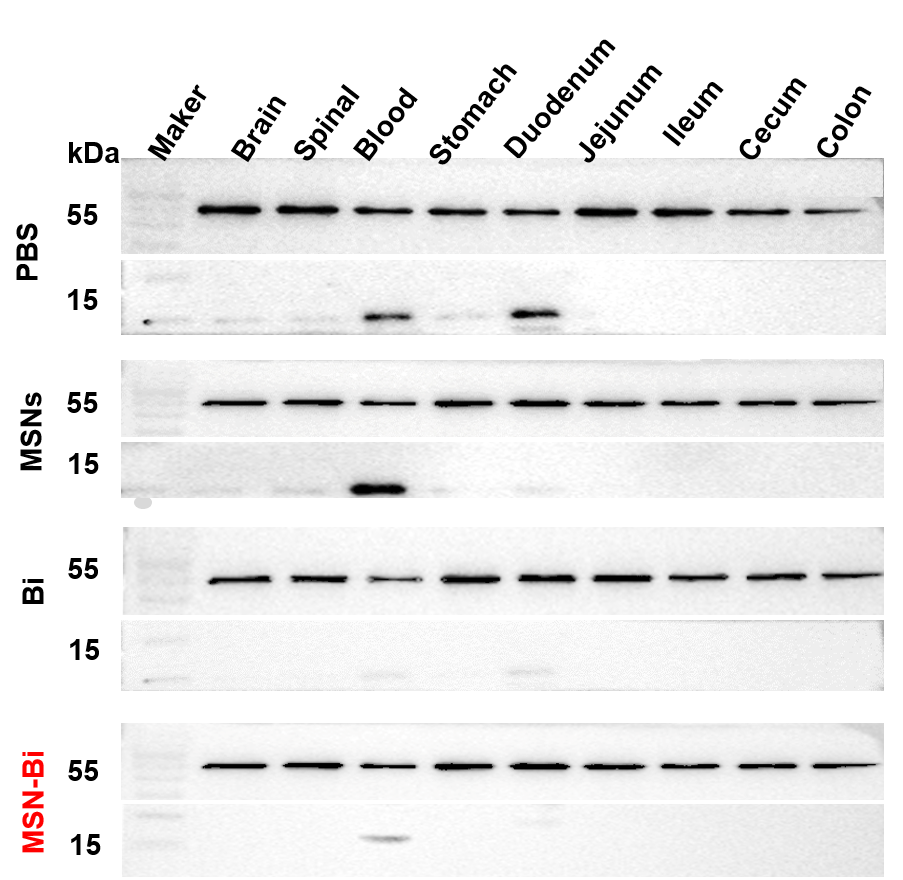
**

**Additional file 1: Figure S9** IP-Western blotting images of the brain, spinal cord, blood, stomach, duodenum, jejunum, ileum, cecum, and colon from APP/PS1 mice treated with PBS, MSNs, *Bifidobacterium*, and MSNs-Bi.

**
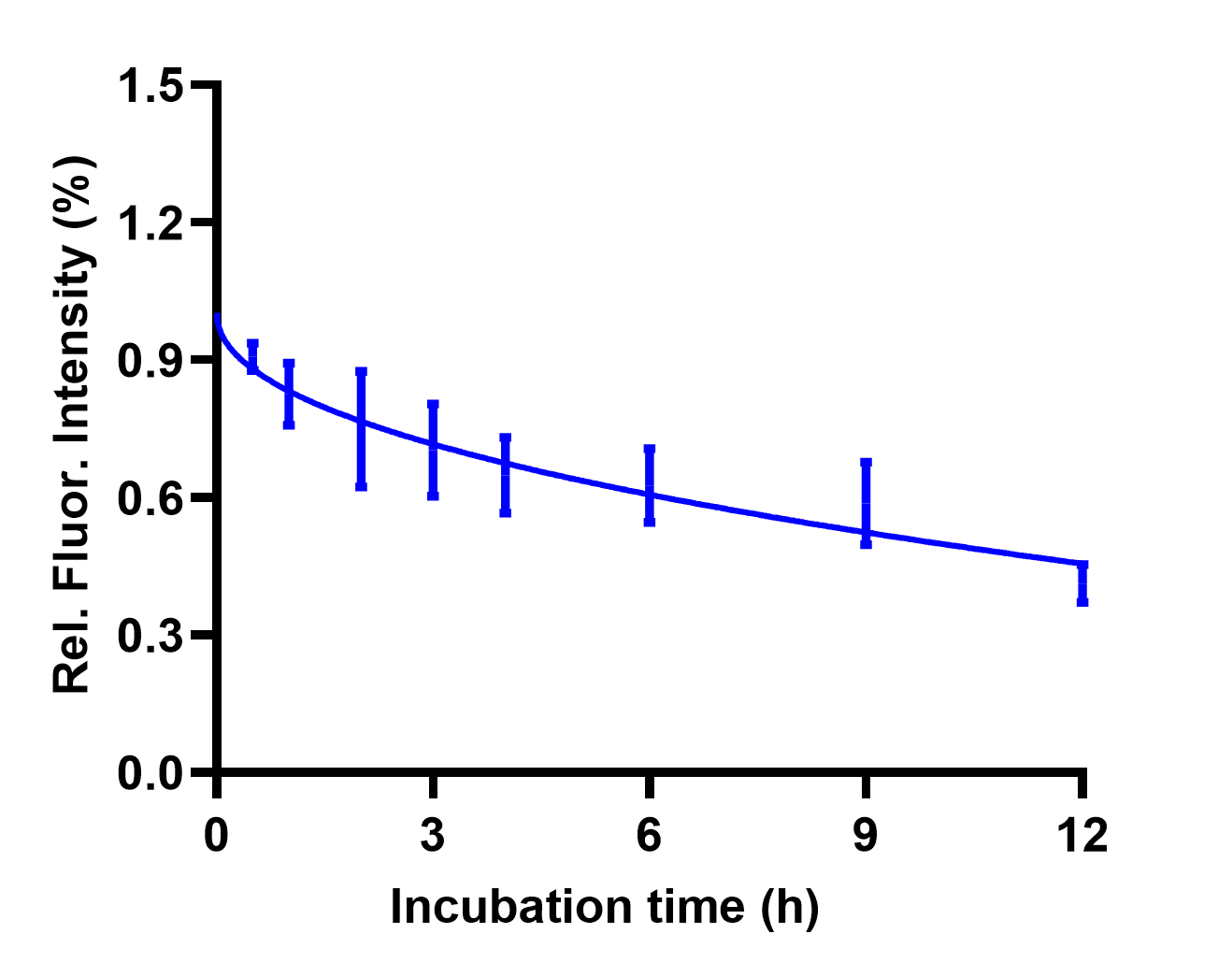
**

**Additional file 1: Figure S10** Relative fluorescence intensity was used to monitor changes in the level of Cy3 in MSNs.
